# Supplementary material for: Using a Mediator's Toolbox: Reducing Clinical Conflict by Learning to Reconceive the “Difficult” Patient or Family
Source: MedEdPORTAL. 2023 Jul 14;19:11324. doi: 10.15766/mep_2374-8265.11324 (PMC10345165; doi:10.15766/mep_2374-8265.11324)
Supplement: Supplementary file 1 — Using the Mediators Toolbox Presentation.pptxView From Everywhere Case Study.docxPositions vs. Interests Case Study.docxWorkshop Evaluation.docx [file mep_2374-8265.11324-s001.zip › A. Using the Mediators Toolbox Presentation.pptx]

## Slide 1
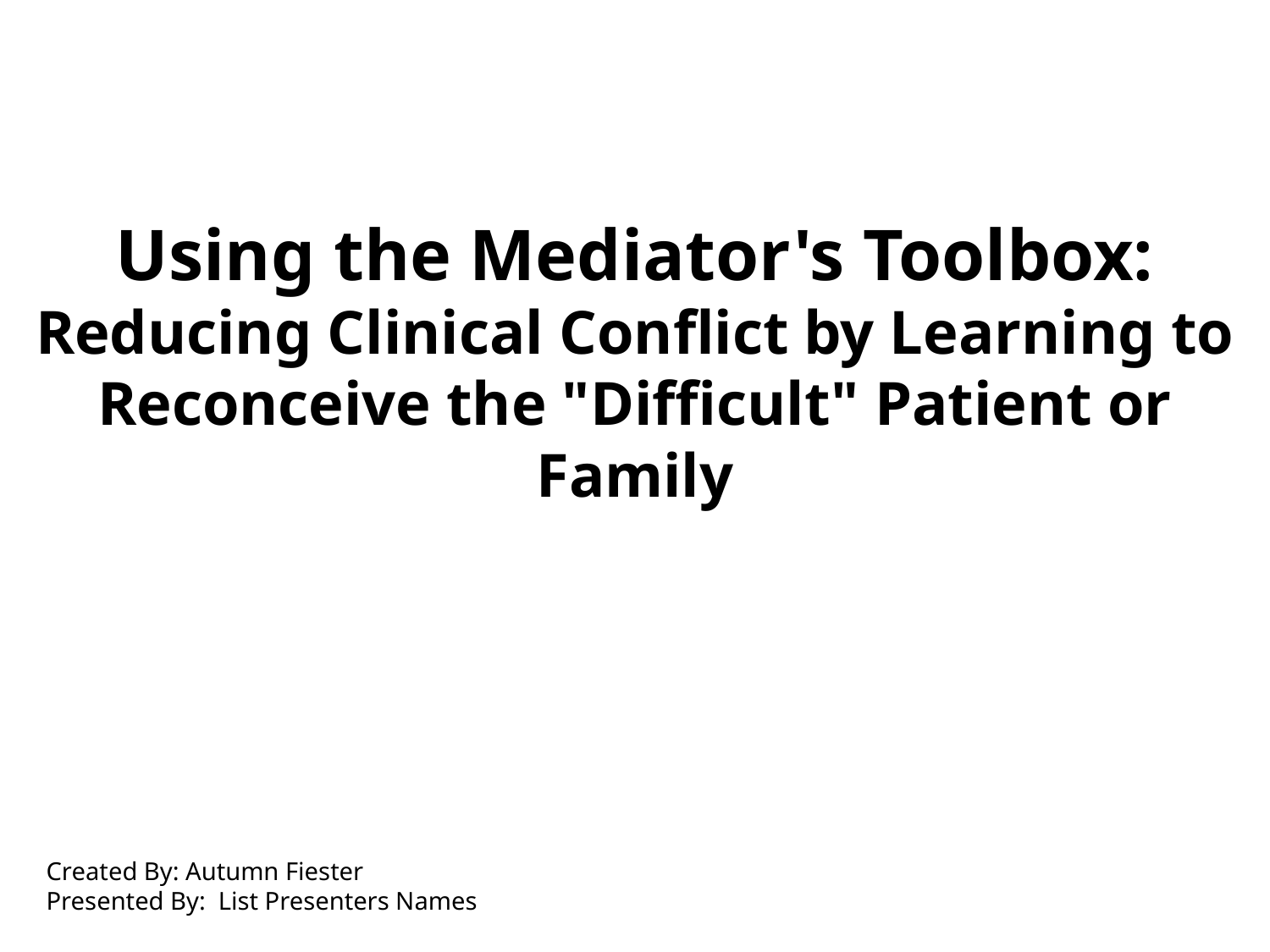

# Using the Mediator's Toolbox: Reducing Clinical Conflict by Learning to Reconceive the "Difficult" Patient or Family
Created By: Autumn Fiester
Presented By: List Presenters Names

## Slide 2
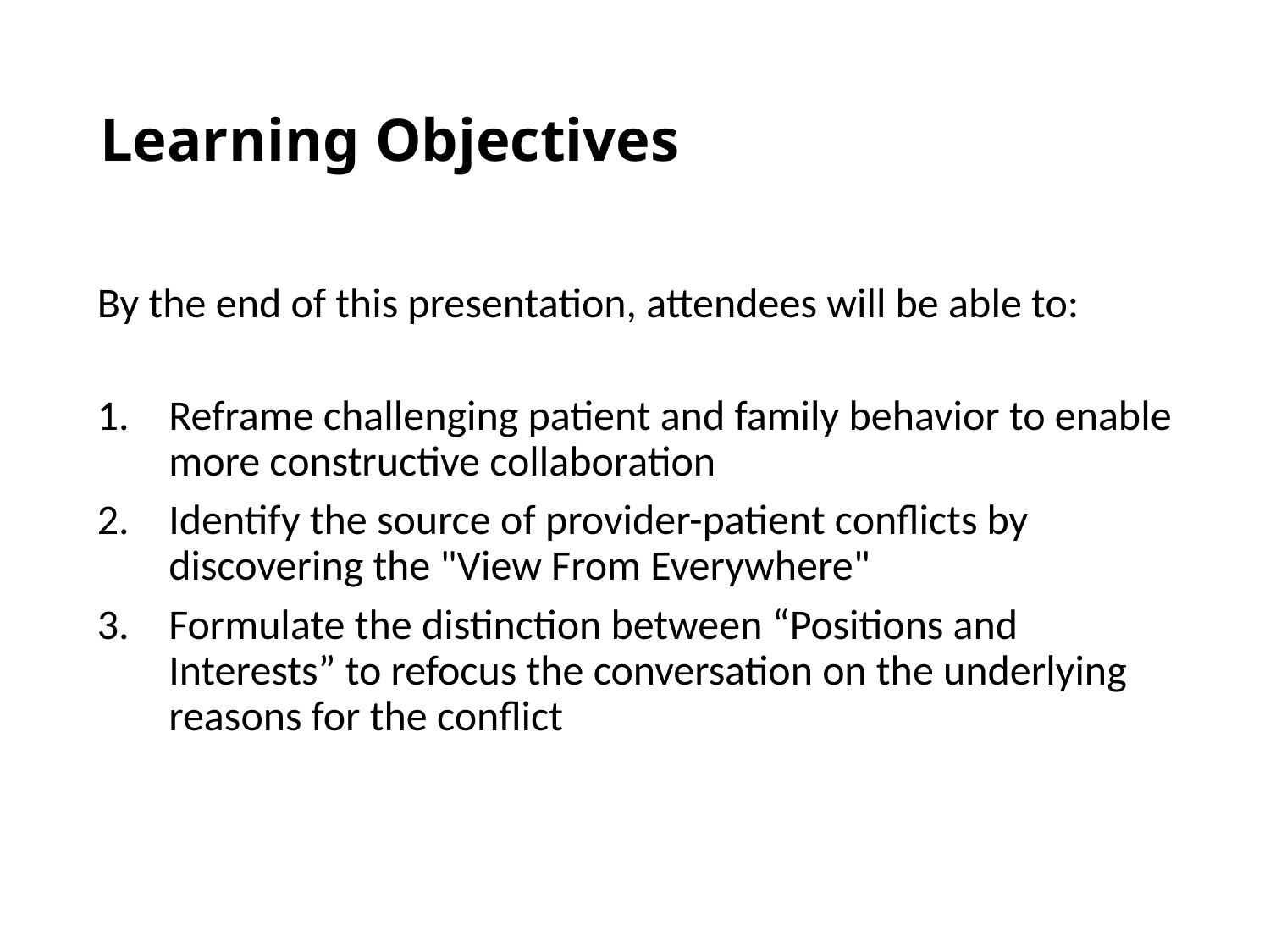

# Learning Objectives
By the end of this presentation, attendees will be able to:
Reframe challenging patient and family behavior to enable more constructive collaboration
Identify the source of provider-patient conflicts by discovering the "View From Everywhere"
Formulate the distinction between “Positions and Interests” to refocus the conversation on the underlying reasons for the conflict

## Slide 3
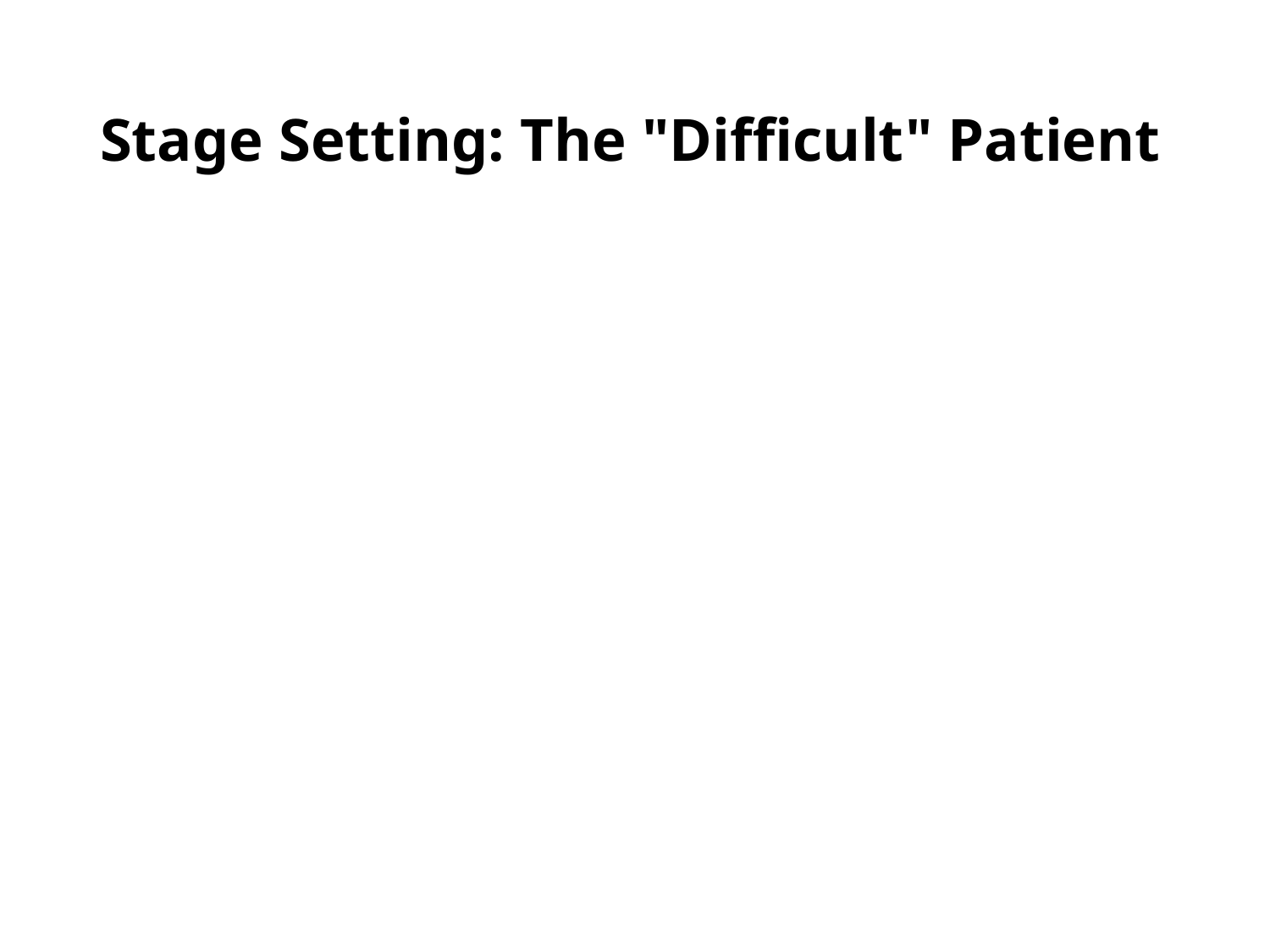

# Stage Setting: The "Difficult" Patient

## Slide 4
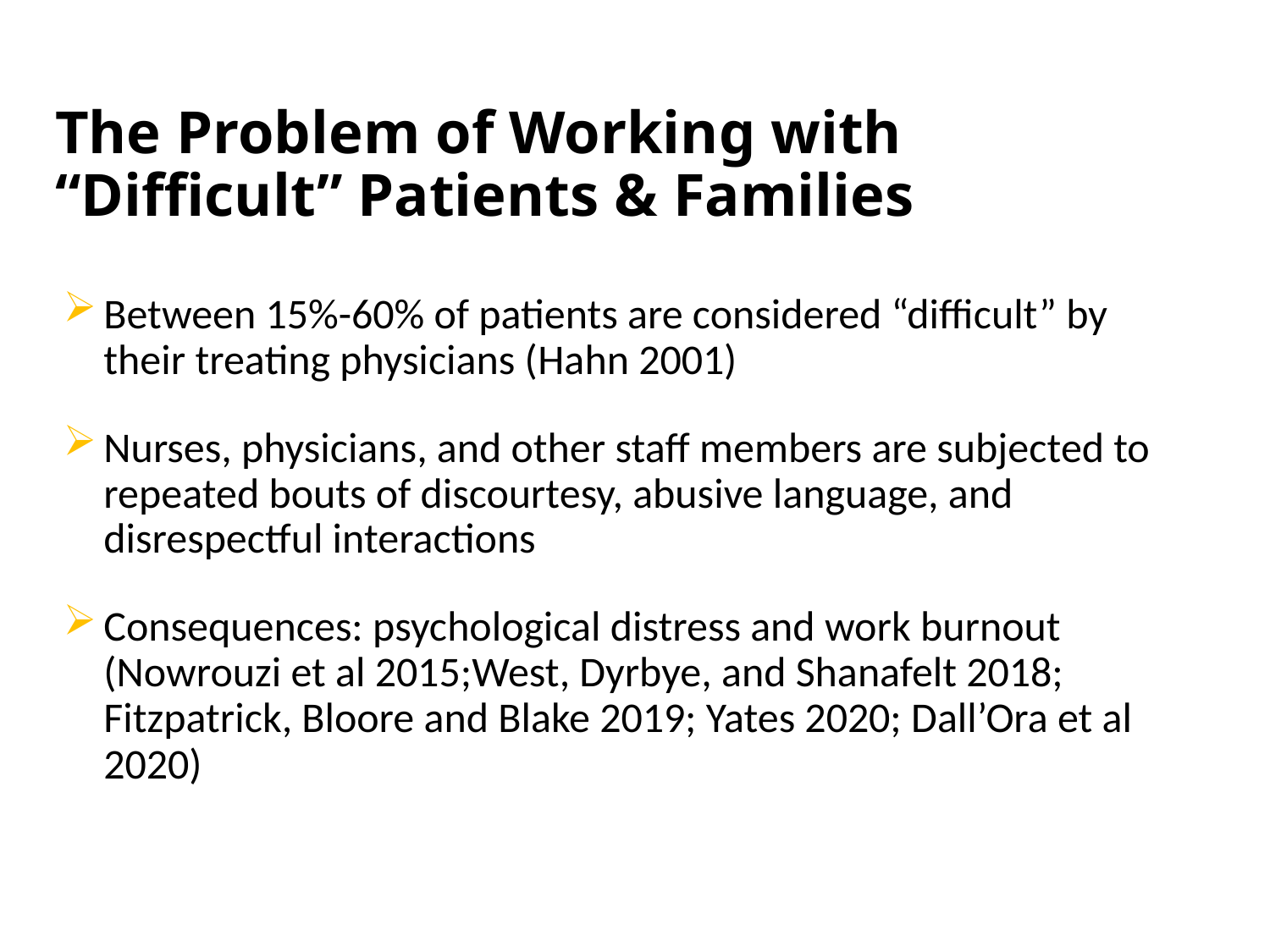

# The Problem of Working with “Difficult” Patients & Families
Between 15%-60% of patients are considered “difficult” by their treating physicians (Hahn 2001)
Nurses, physicians, and other staff members are subjected to repeated bouts of discourtesy, abusive language, and disrespectful interactions
Consequences: psychological distress and work burnout (Nowrouzi et al 2015;West, Dyrbye, and Shanafelt 2018; Fitzpatrick, Bloore and Blake 2019; Yates 2020; Dall’Ora et al 2020)

## Slide 5
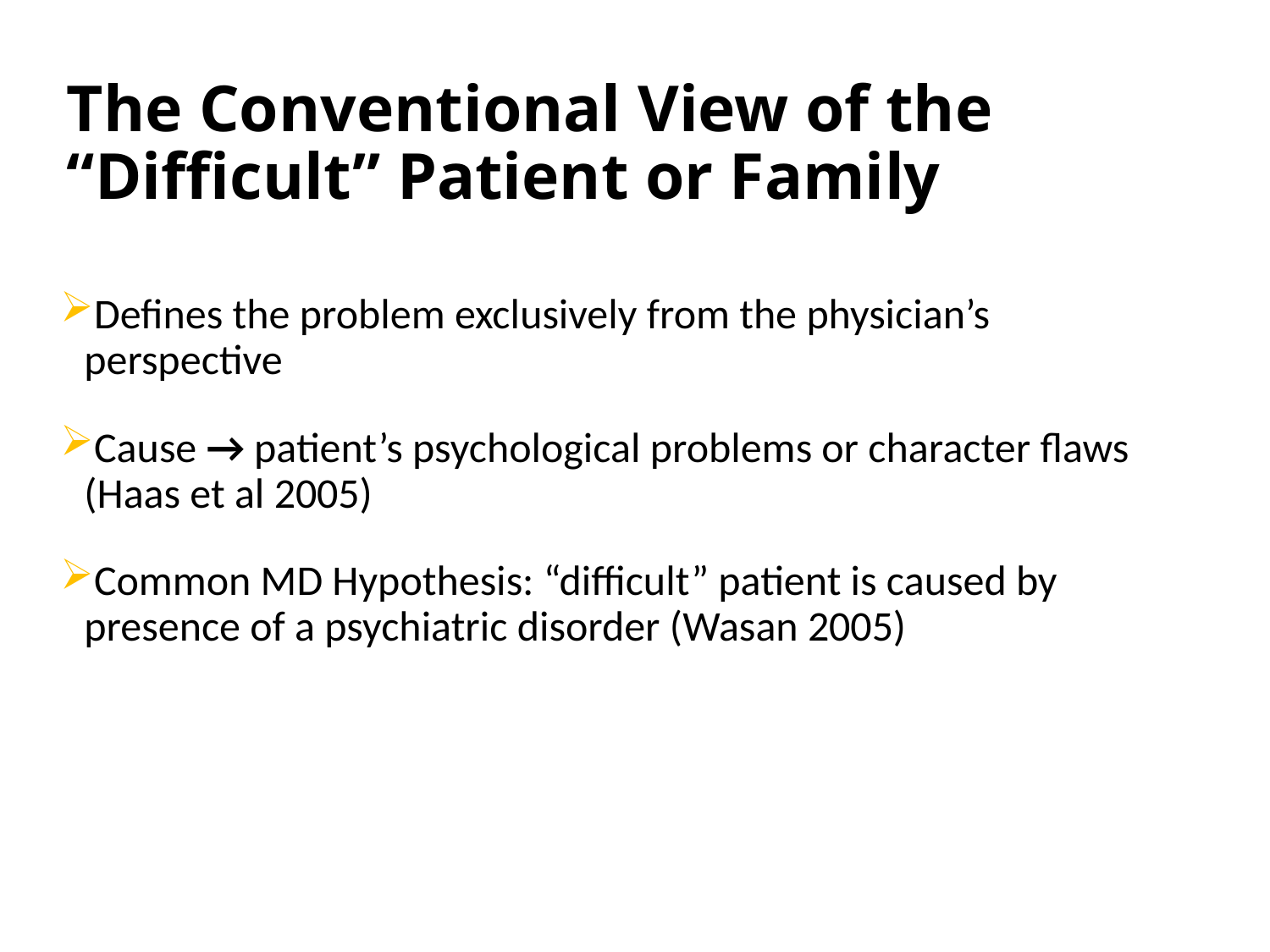

# The Conventional View of the “Difficult” Patient or Family
Defines the problem exclusively from the physician’s perspective
Cause → patient’s psychological problems or character flaws (Haas et al 2005)
Common MD Hypothesis: “difficult” patient is caused by presence of a psychiatric disorder (Wasan 2005)

## Slide 6
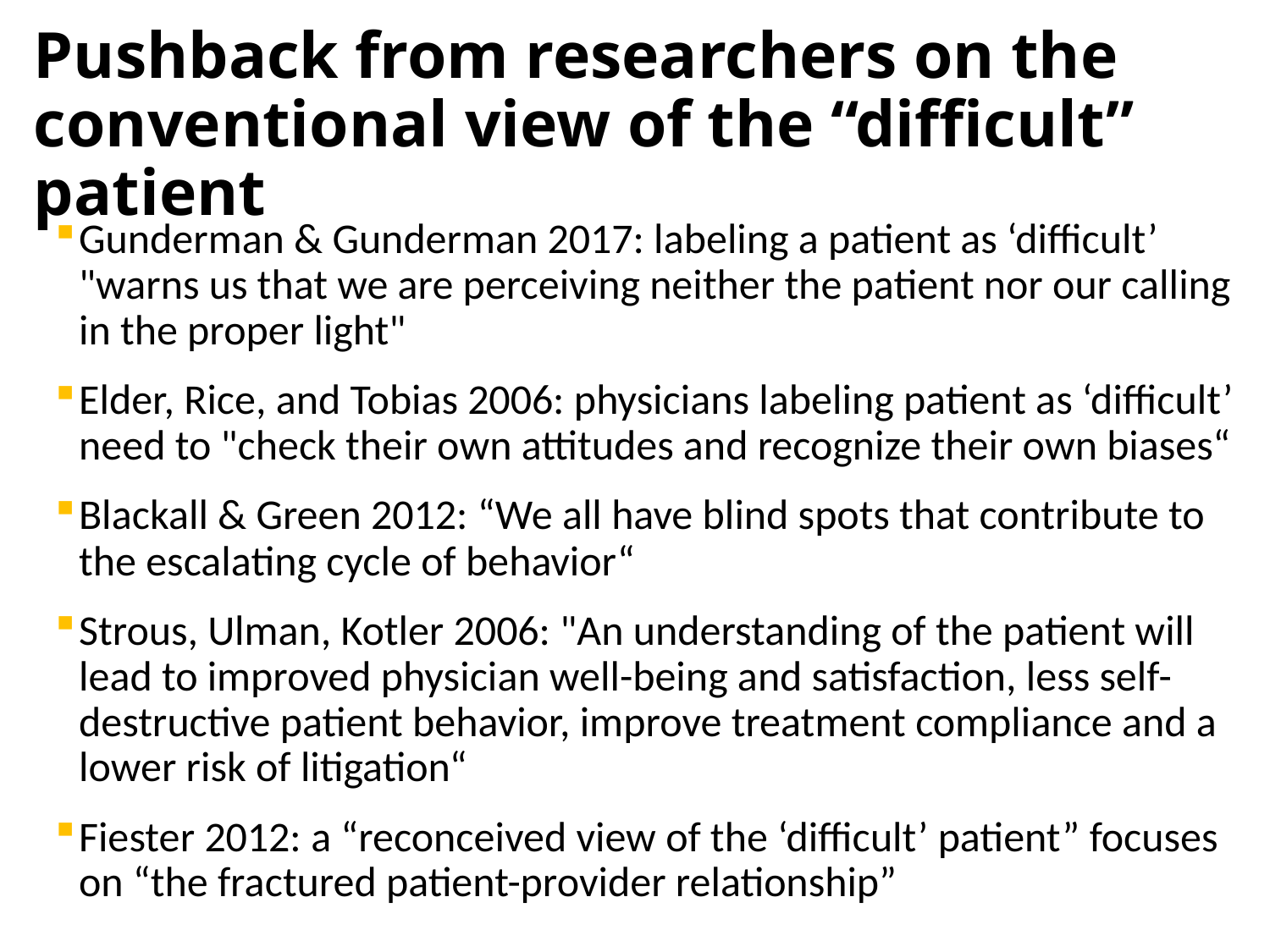

# Pushback from researchers on the conventional view of the “difficult” patient
Gunderman & Gunderman 2017: labeling a patient as ‘difficult’ "warns us that we are perceiving neither the patient nor our calling in the proper light"
Elder, Rice, and Tobias 2006: physicians labeling patient as ‘difficult’ need to "check their own attitudes and recognize their own biases“
Blackall & Green 2012: “We all have blind spots that contribute to the escalating cycle of behavior“
Strous, Ulman, Kotler 2006: "An understanding of the patient will lead to improved physician well-being and satisfaction, less self-destructive patient behavior, improve treatment compliance and a lower risk of litigation“
Fiester 2012: a “reconceived view of the ‘difficult’ patient” focuses on “the fractured patient-provider relationship”

## Slide 7
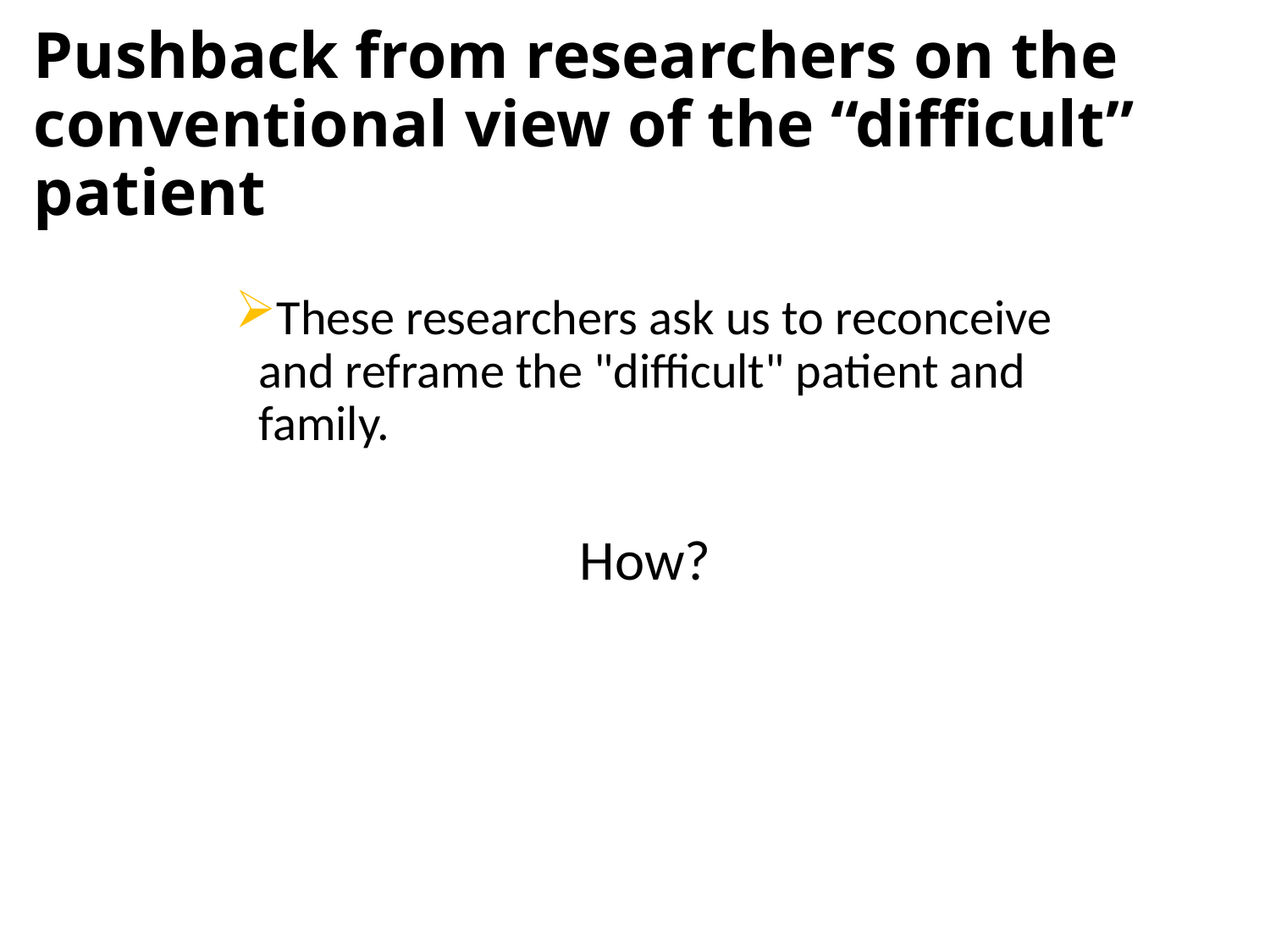

# Pushback from researchers on the conventional view of the “difficult” patient
These researchers ask us to reconceive and reframe the "difficult" patient and family.
How?

## Slide 8
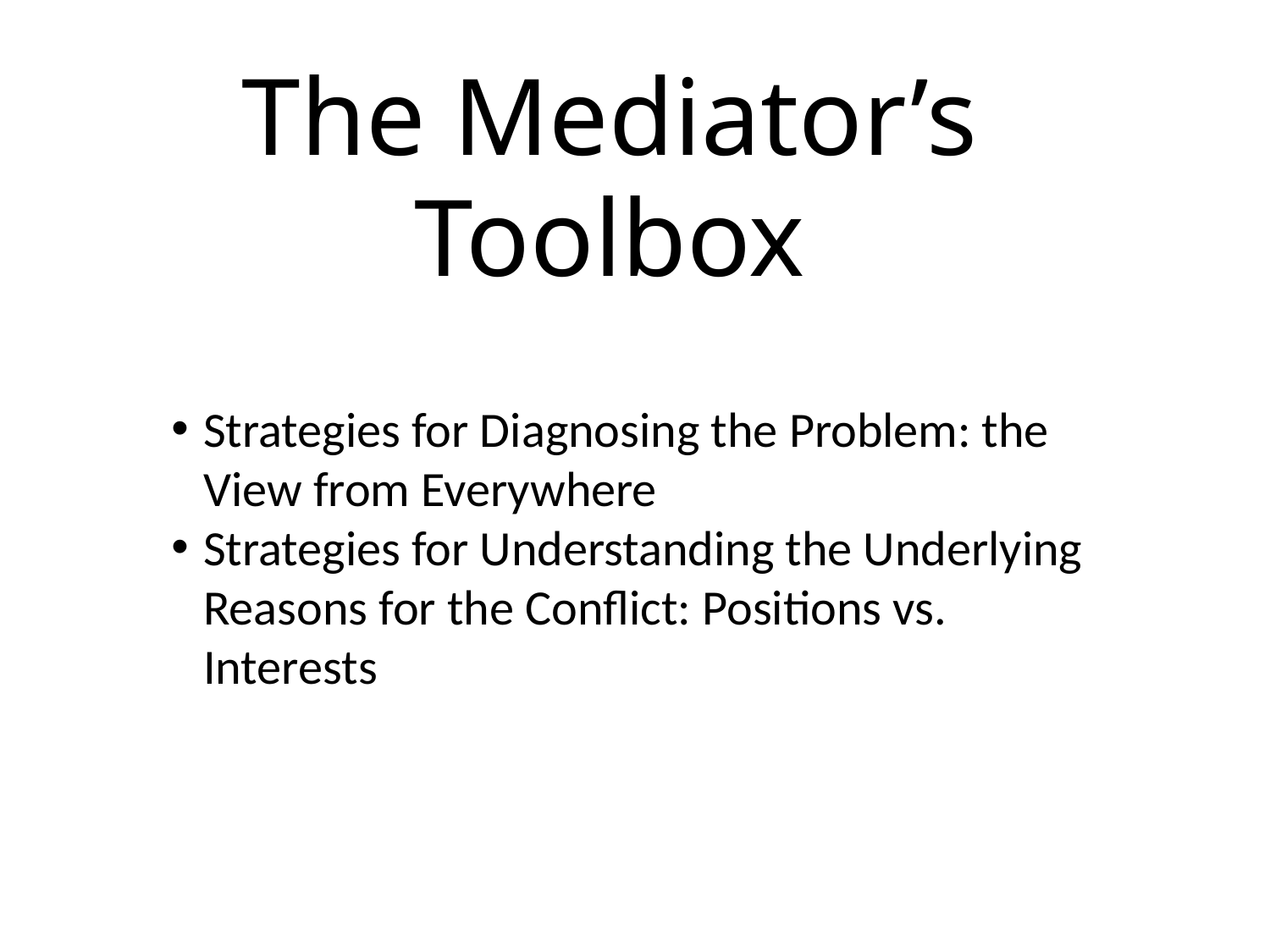

# The Mediator’s Toolbox
Strategies for Diagnosing the Problem: the View from Everywhere
Strategies for Understanding the Underlying Reasons for the Conflict: Positions vs. Interests

## Slide 9
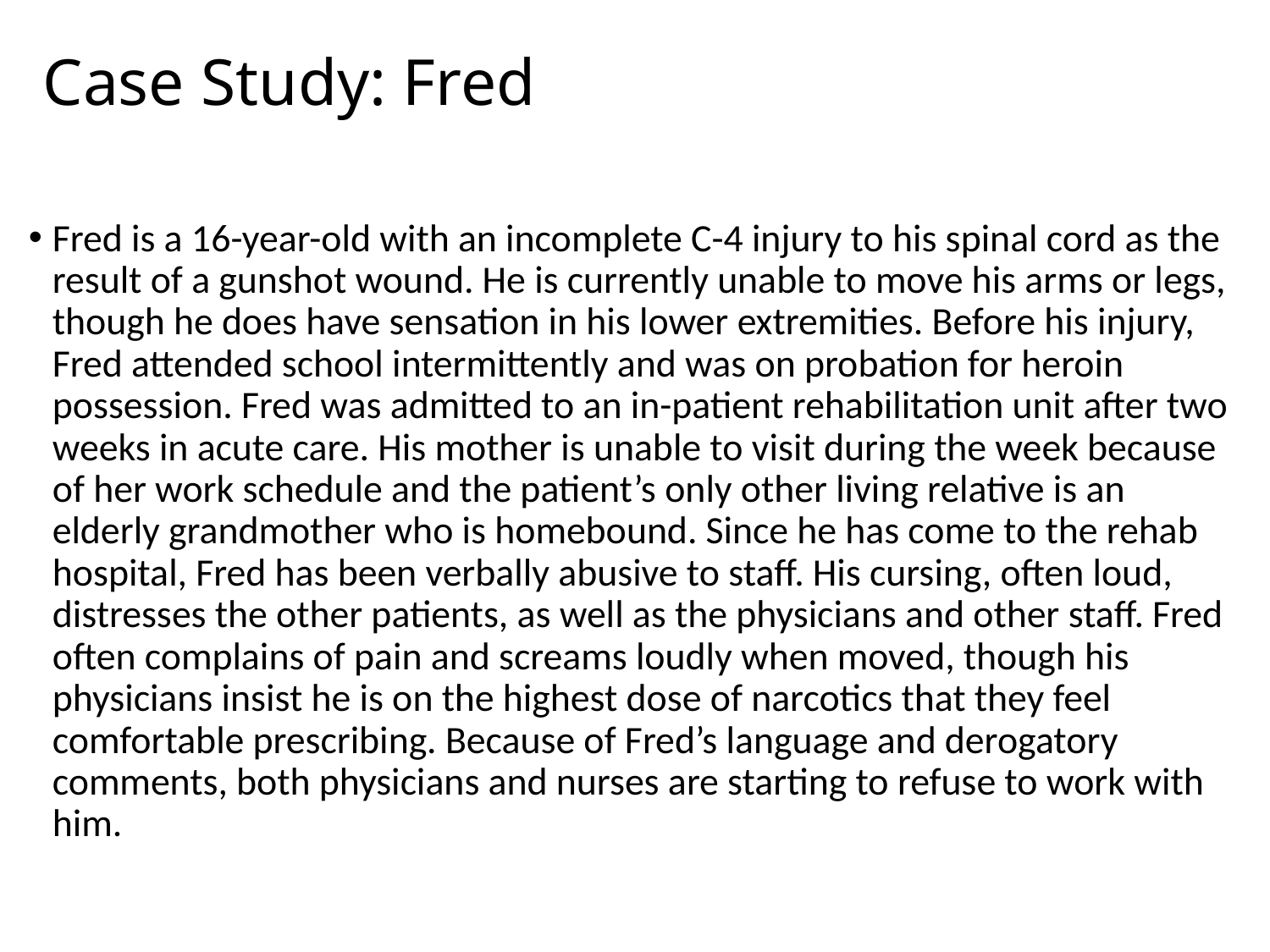

# Case Study: Fred
Fred is a 16-year-old with an incomplete C-4 injury to his spinal cord as the result of a gunshot wound. He is currently unable to move his arms or legs, though he does have sensation in his lower extremities. Before his injury, Fred attended school intermittently and was on probation for heroin possession. Fred was admitted to an in-patient rehabilitation unit after two weeks in acute care. His mother is unable to visit during the week because of her work schedule and the patient’s only other living relative is an elderly grandmother who is homebound. Since he has come to the rehab hospital, Fred has been verbally abusive to staff. His cursing, often loud, distresses the other patients, as well as the physicians and other staff. Fred often complains of pain and screams loudly when moved, though his physicians insist he is on the highest dose of narcotics that they feel comfortable prescribing. Because of Fred’s language and derogatory comments, both physicians and nurses are starting to refuse to work with him.

## Slide 10
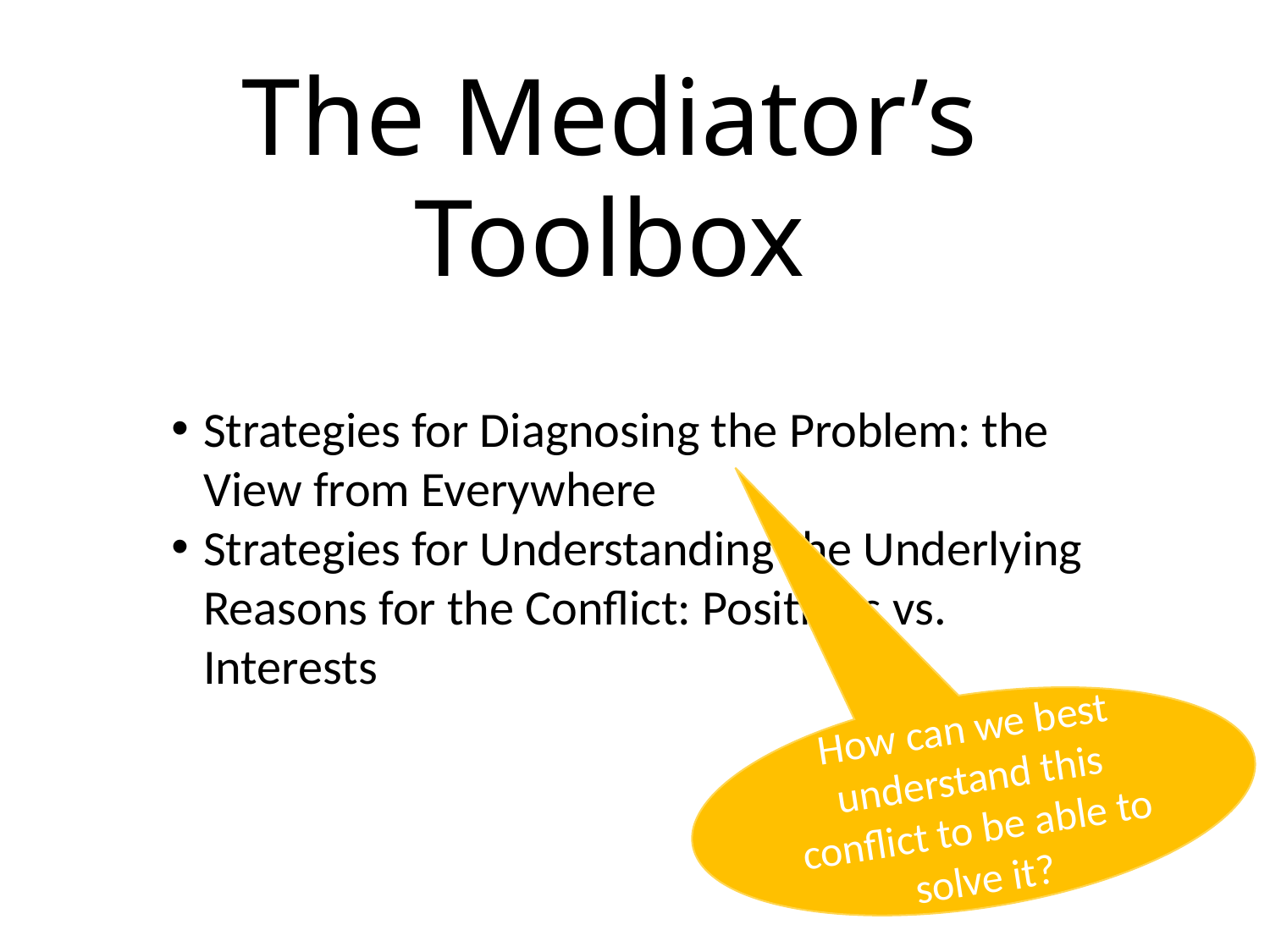

# The Mediator’s Toolbox
Strategies for Diagnosing the Problem: the View from Everywhere
Strategies for Understanding the Underlying Reasons for the Conflict: Positions vs. Interests
How can we best understand this conflict to be able to solve it?

## Slide 11
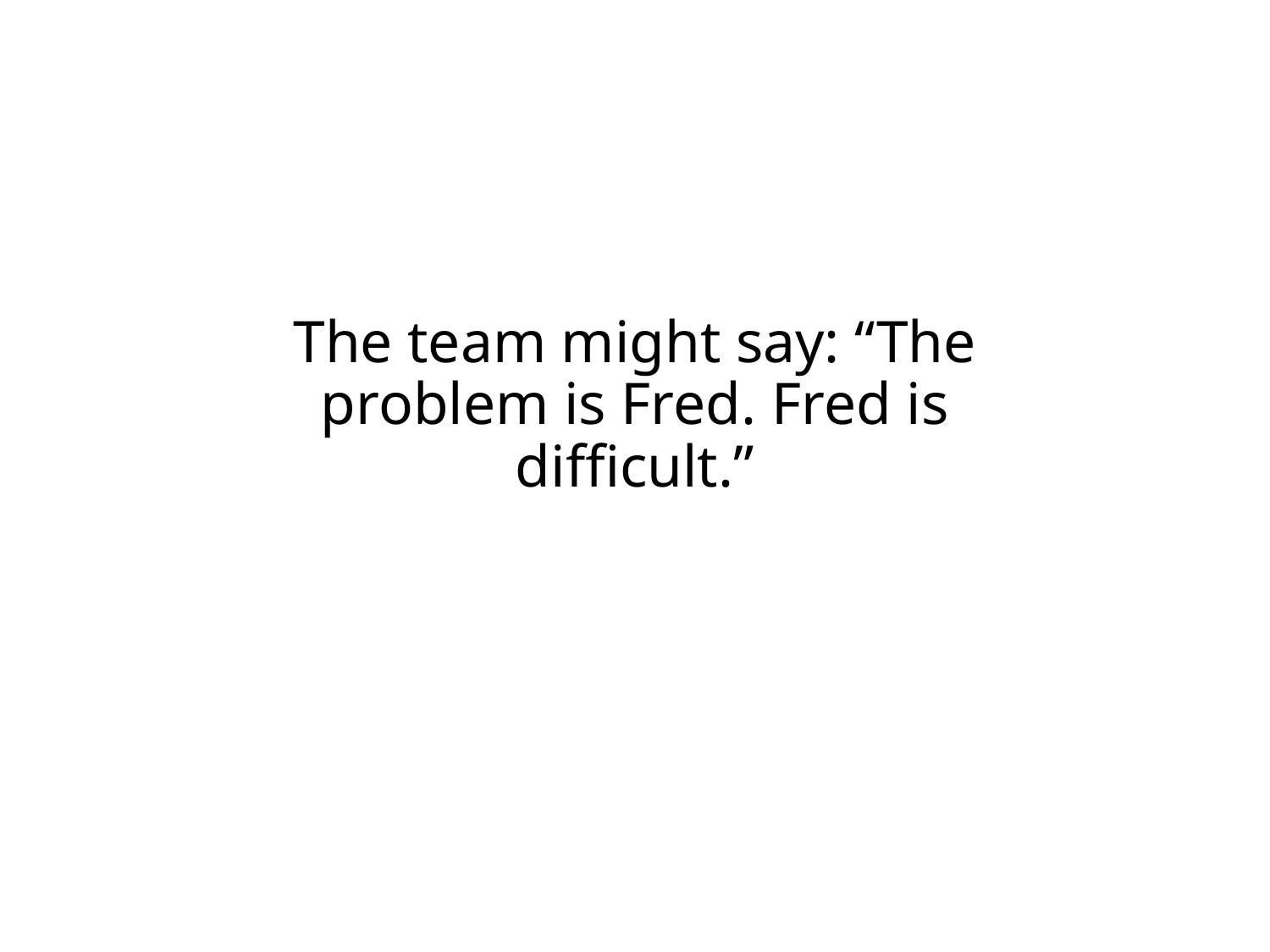

# The team might say: “The problem is Fred. Fred is difficult.”

## Slide 12
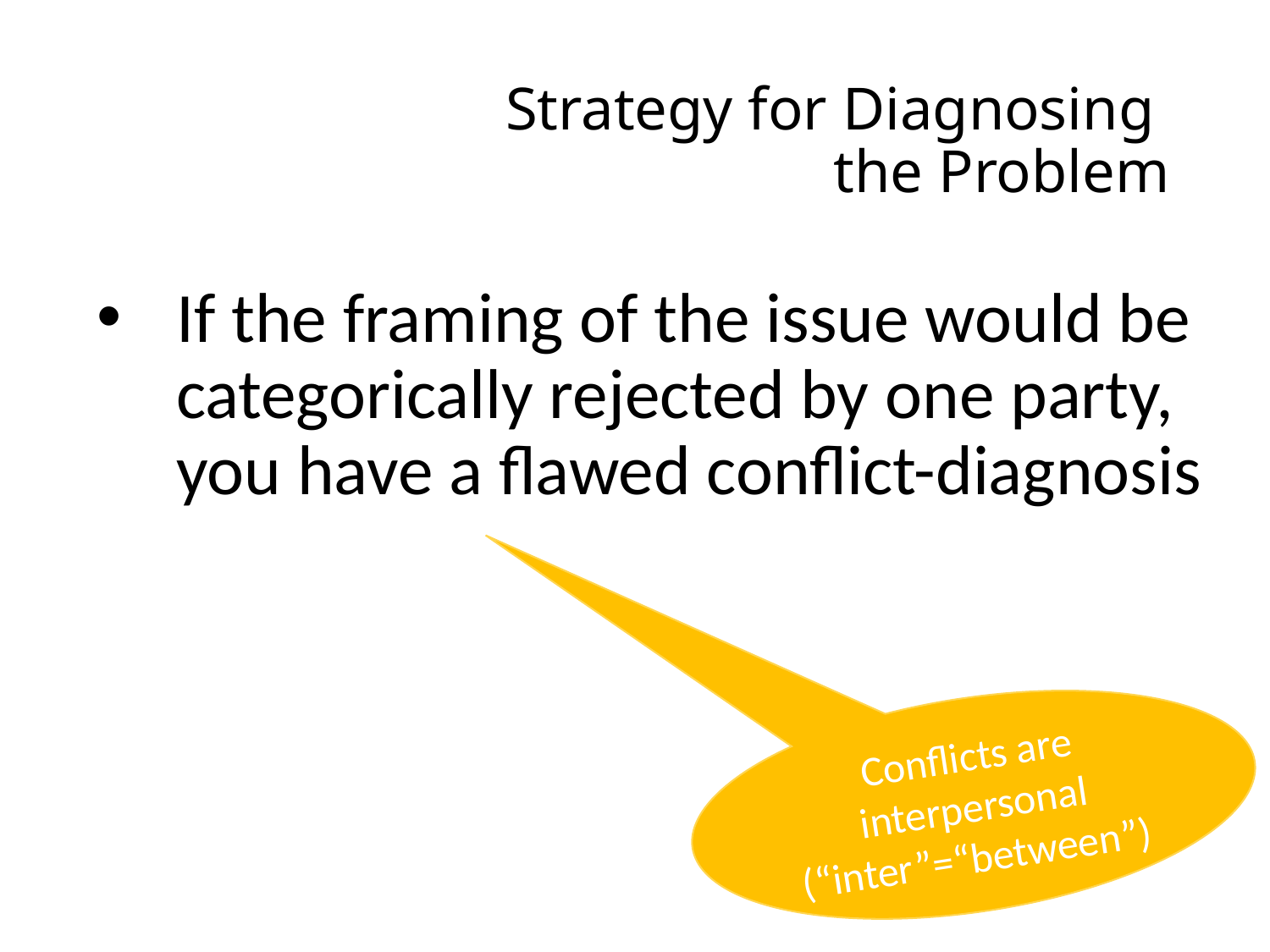

# Strategy for Diagnosing the Problem
If the framing of the issue would be categorically rejected by one party, you have a flawed conflict-diagnosis
Conflicts are interpersonal (“inter”=“between”)

## Slide 13
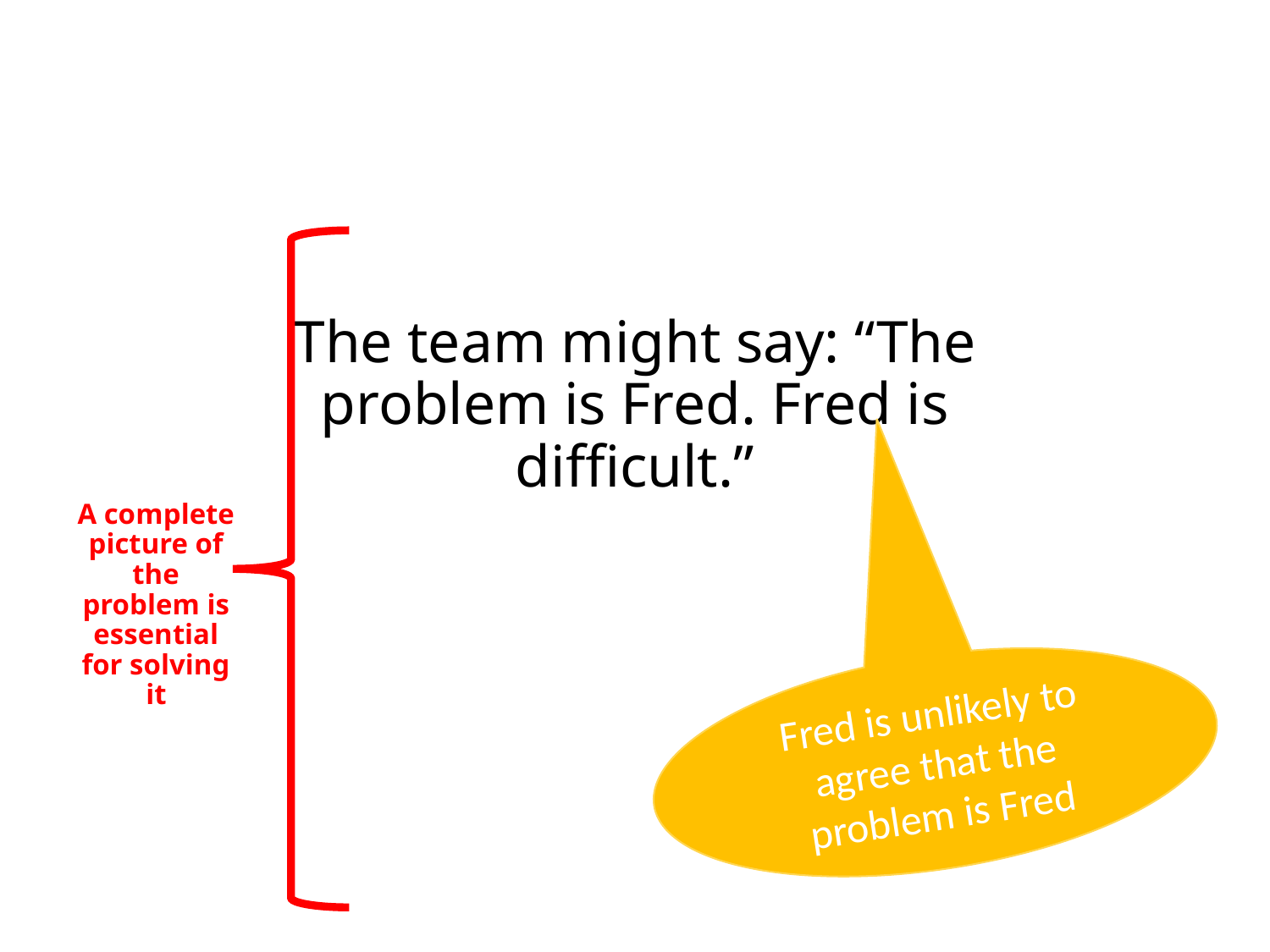

# The team might say: “The problem is Fred. Fred is difficult.”
A complete picture of the problem is essential for solving it
Fred is unlikely to agree that the problem is Fred

## Slide 14
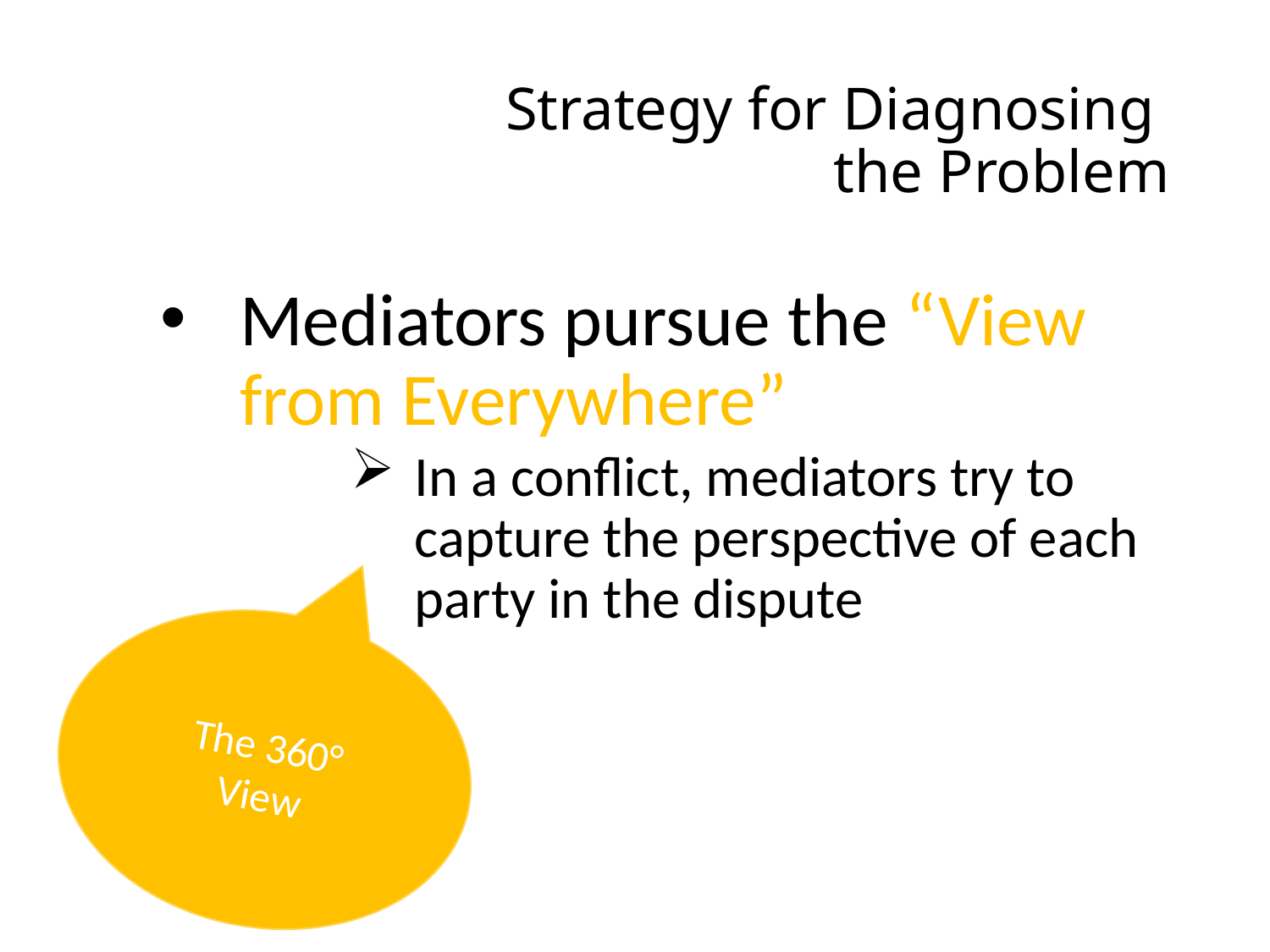

# Strategy for Diagnosing the Problem
Mediators pursue the “View from Everywhere”
In a conflict, mediators try to capture the perspective of each party in the dispute
The 360°
View

## Slide 15
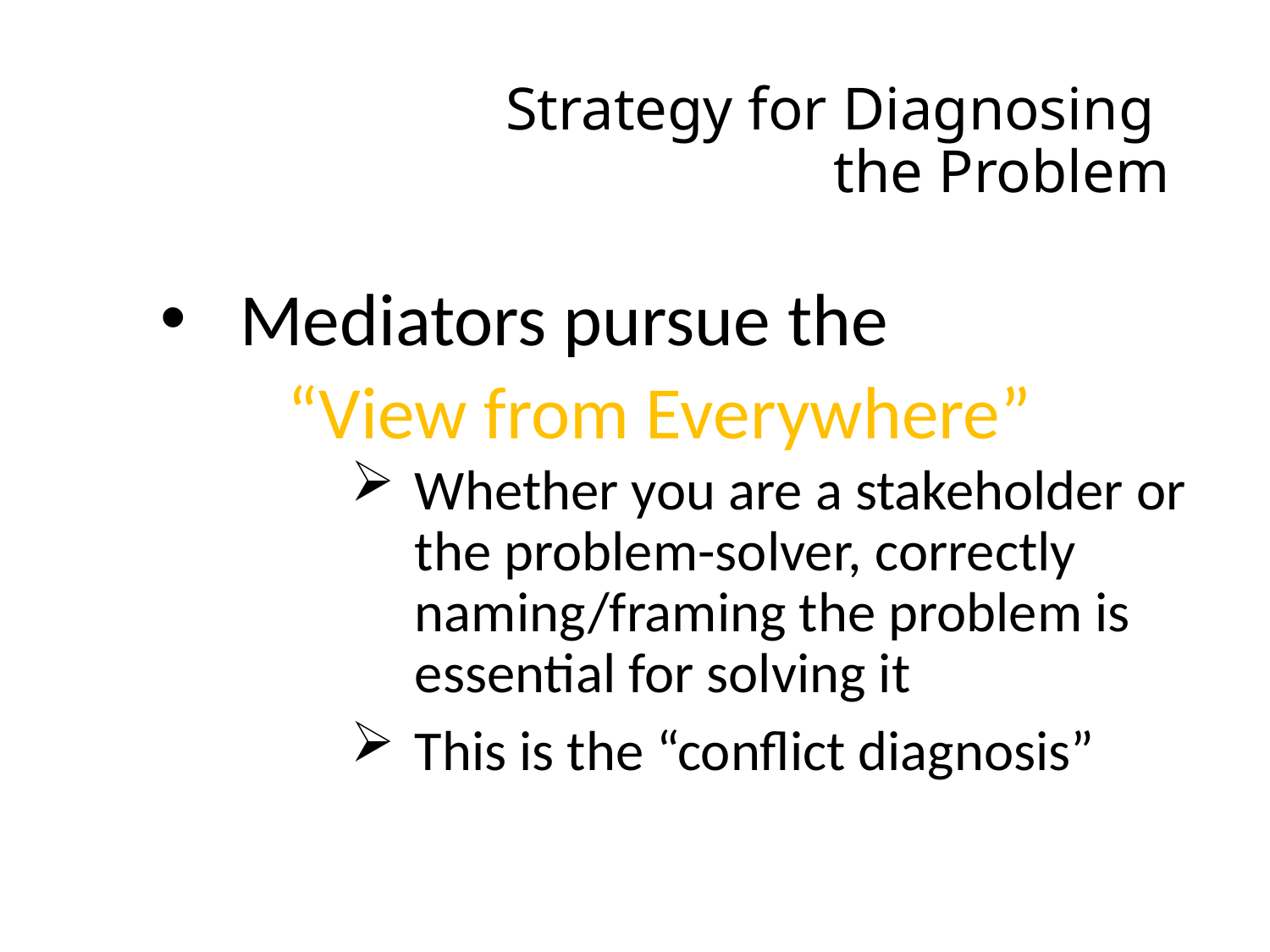

# Strategy for Diagnosing the Problem
Mediators pursue the
	“View from Everywhere”
Whether you are a stakeholder or the problem-solver, correctly naming/framing the problem is essential for solving it
This is the “conflict diagnosis”

## Slide 16
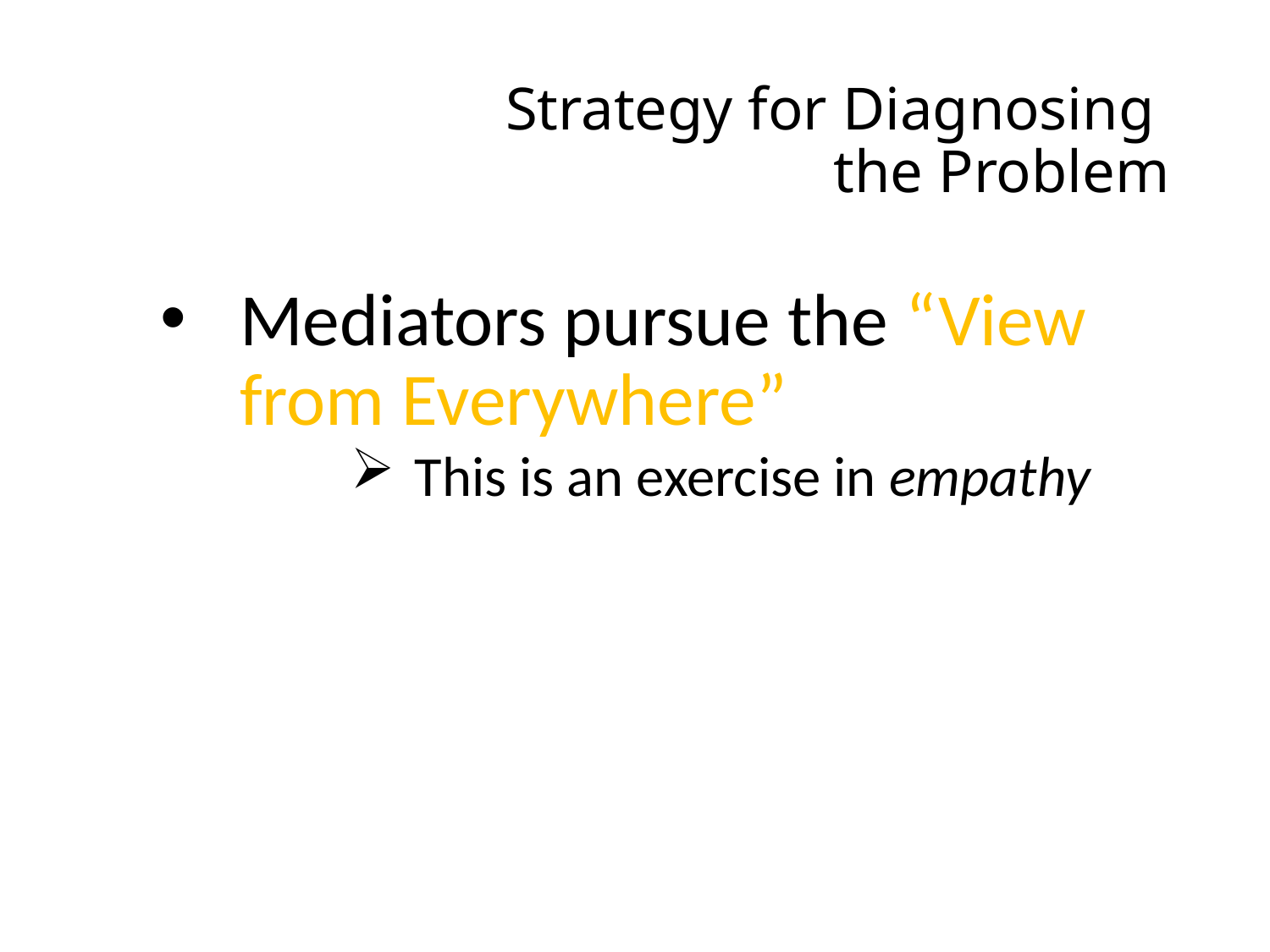

# Strategy for Diagnosing the Problem
Mediators pursue the “View from Everywhere”
This is an exercise in empathy

## Slide 17
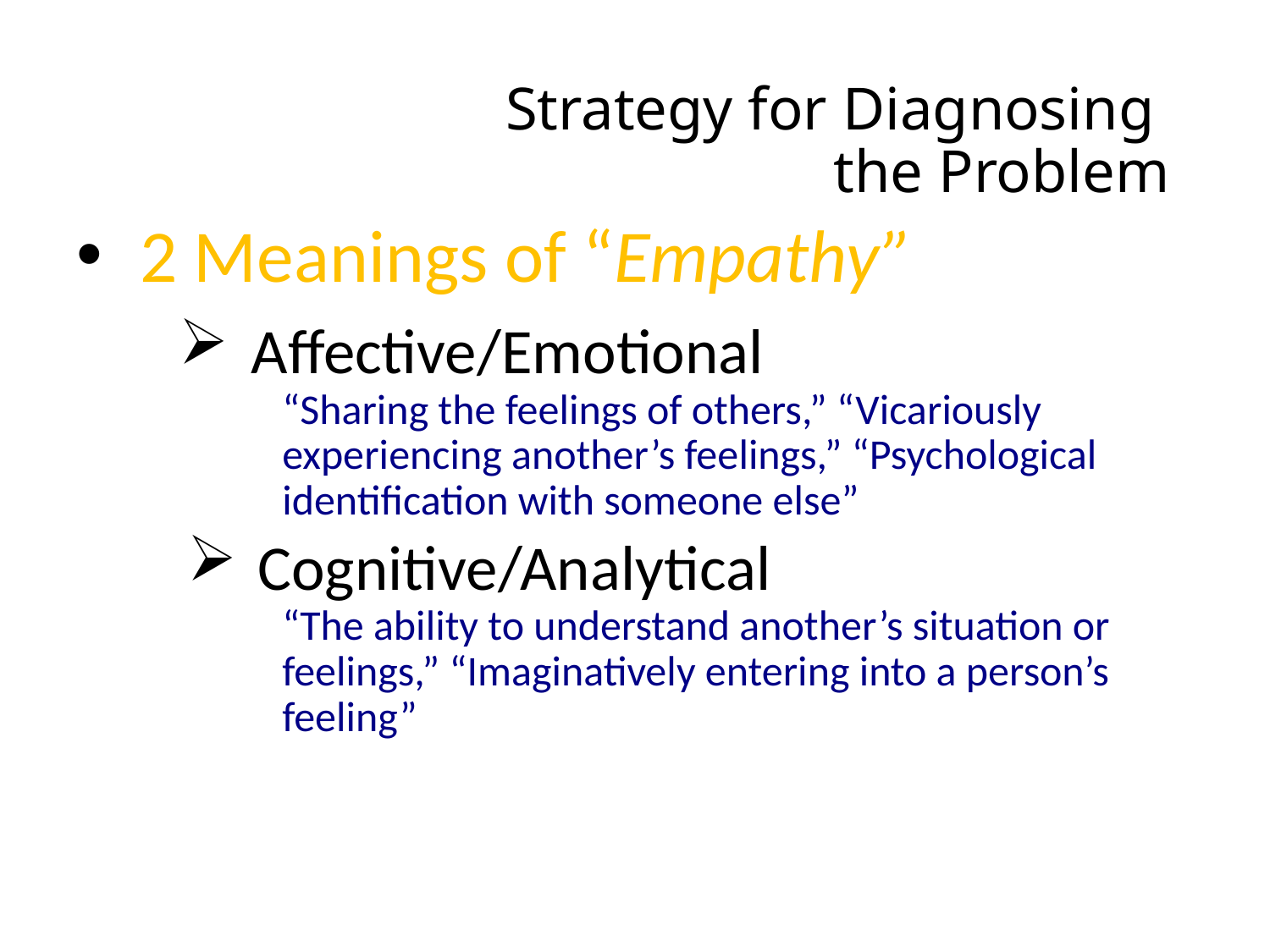

# Strategy for Diagnosing the Problem
2 Meanings of “Empathy”
Affective/Emotional
“Sharing the feelings of others,” “Vicariously experiencing another’s feelings,” “Psychological identification with someone else”
Cognitive/Analytical
“The ability to understand another’s situation or feelings,” “Imaginatively entering into a person’s feeling”

## Slide 18
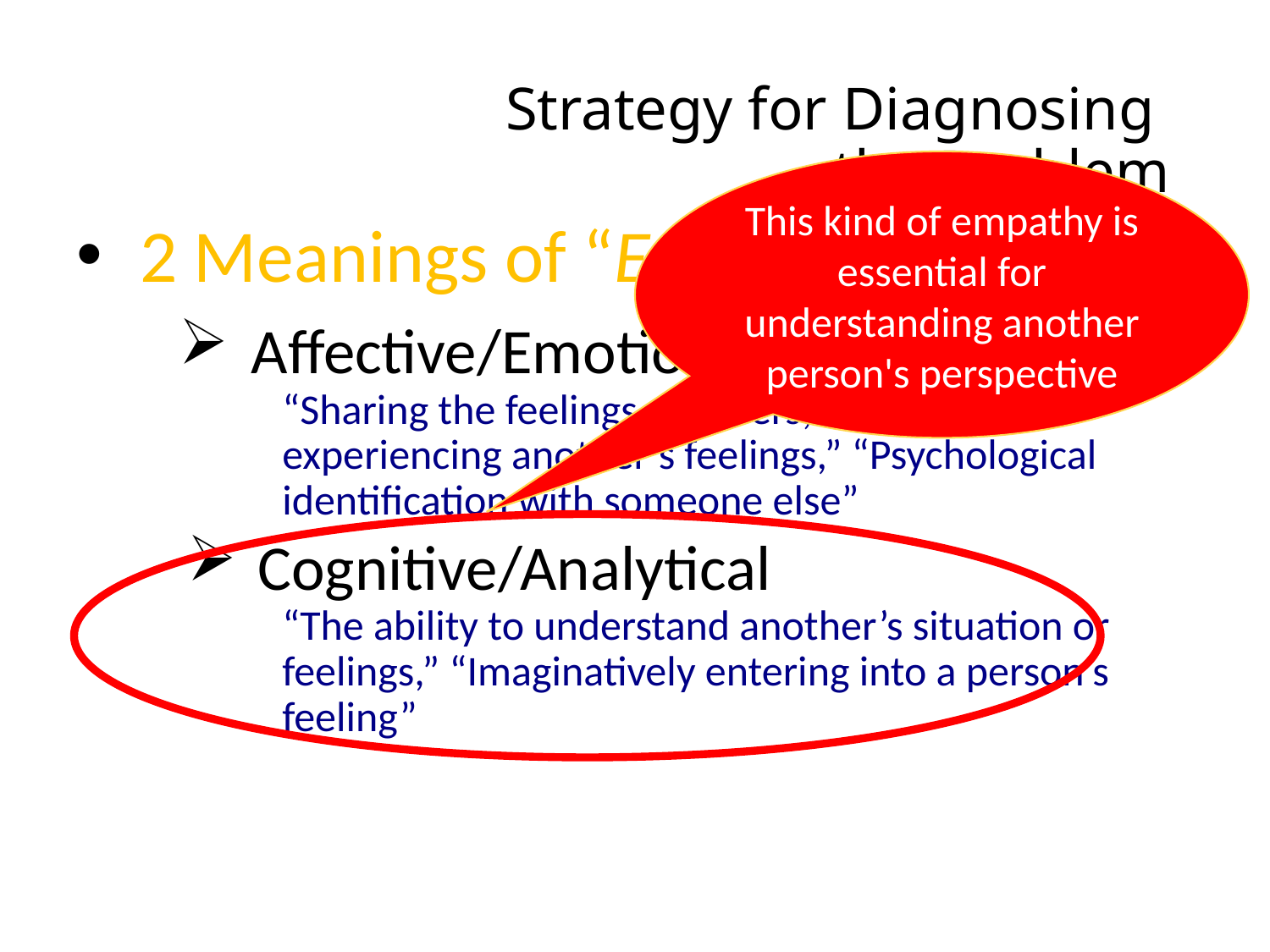

# Strategy for Diagnosing the Problem
This kind of empathy is essential for understanding another person's perspective
2 Meanings of “Empathy”
Affective/Emotional
“Sharing the feelings of others,” “Vicariously experiencing another’s feelings,” “Psychological identification with someone else”
Cognitive/Analytical
“The ability to understand another’s situation or feelings,” “Imaginatively entering into a person’s feeling”

## Slide 19
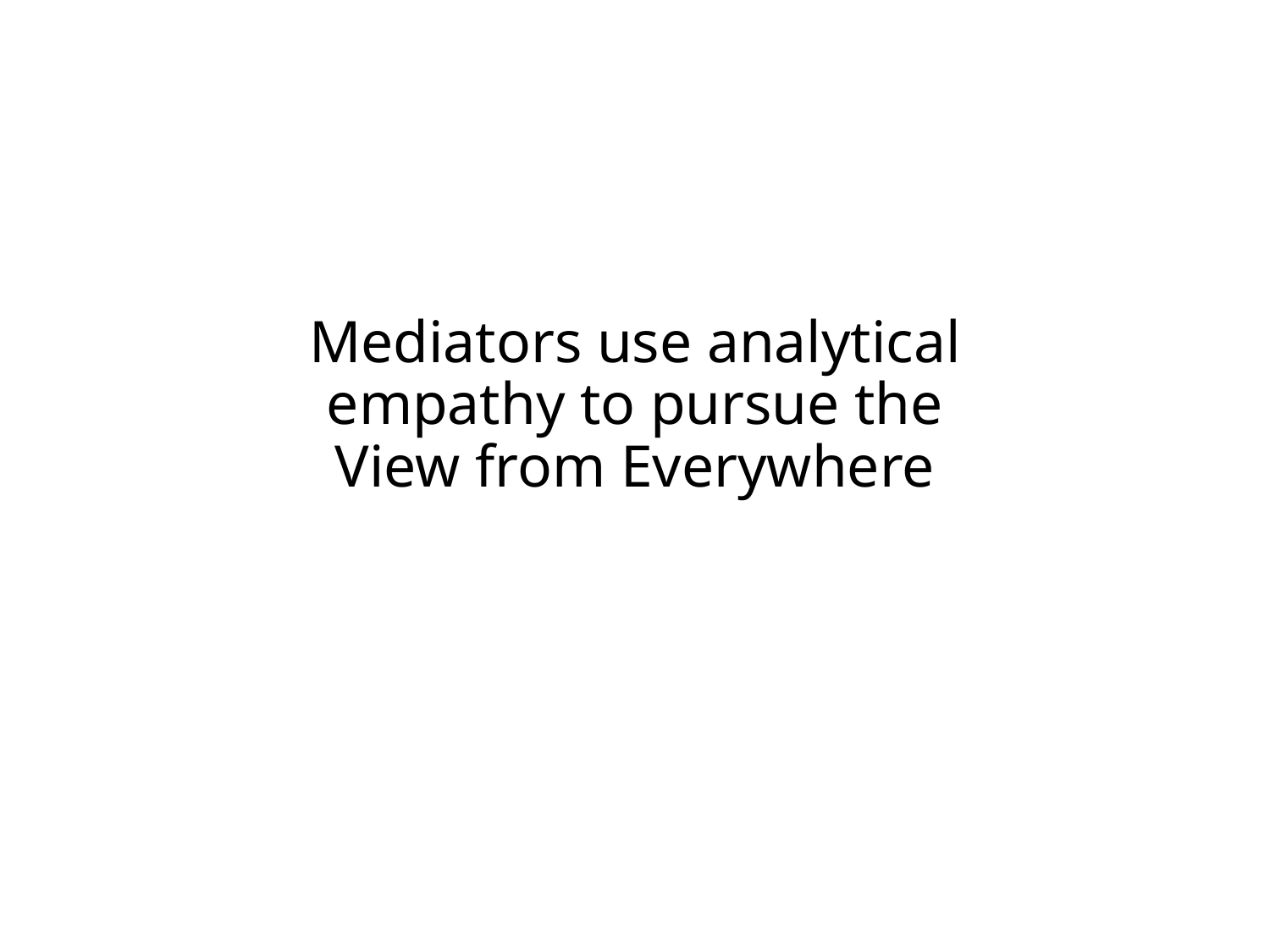

# Mediators use analytical empathy to pursue the View from Everywhere

## Slide 20
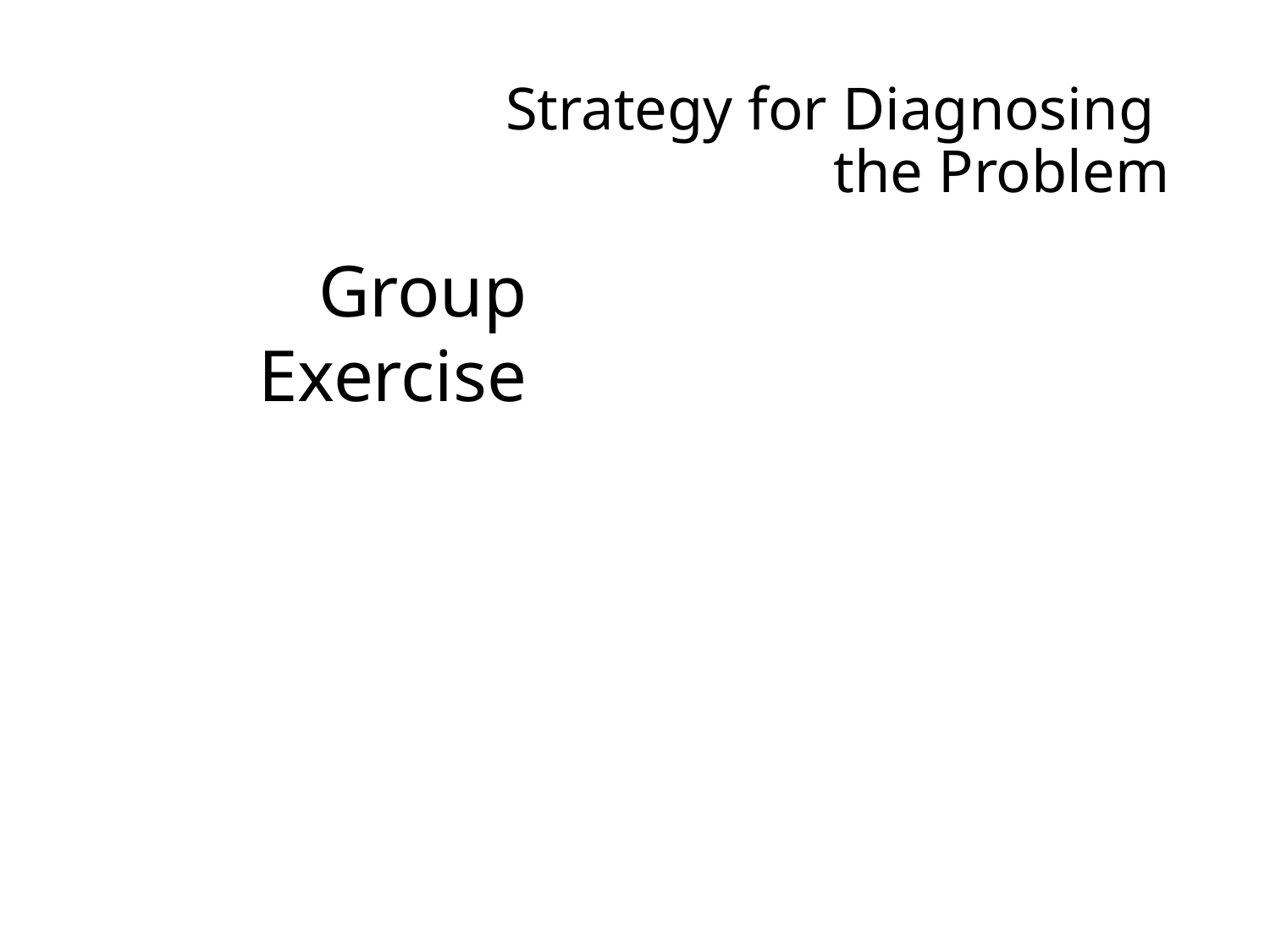

# Strategy for Diagnosing the Problem
Group Exercise

## Slide 21
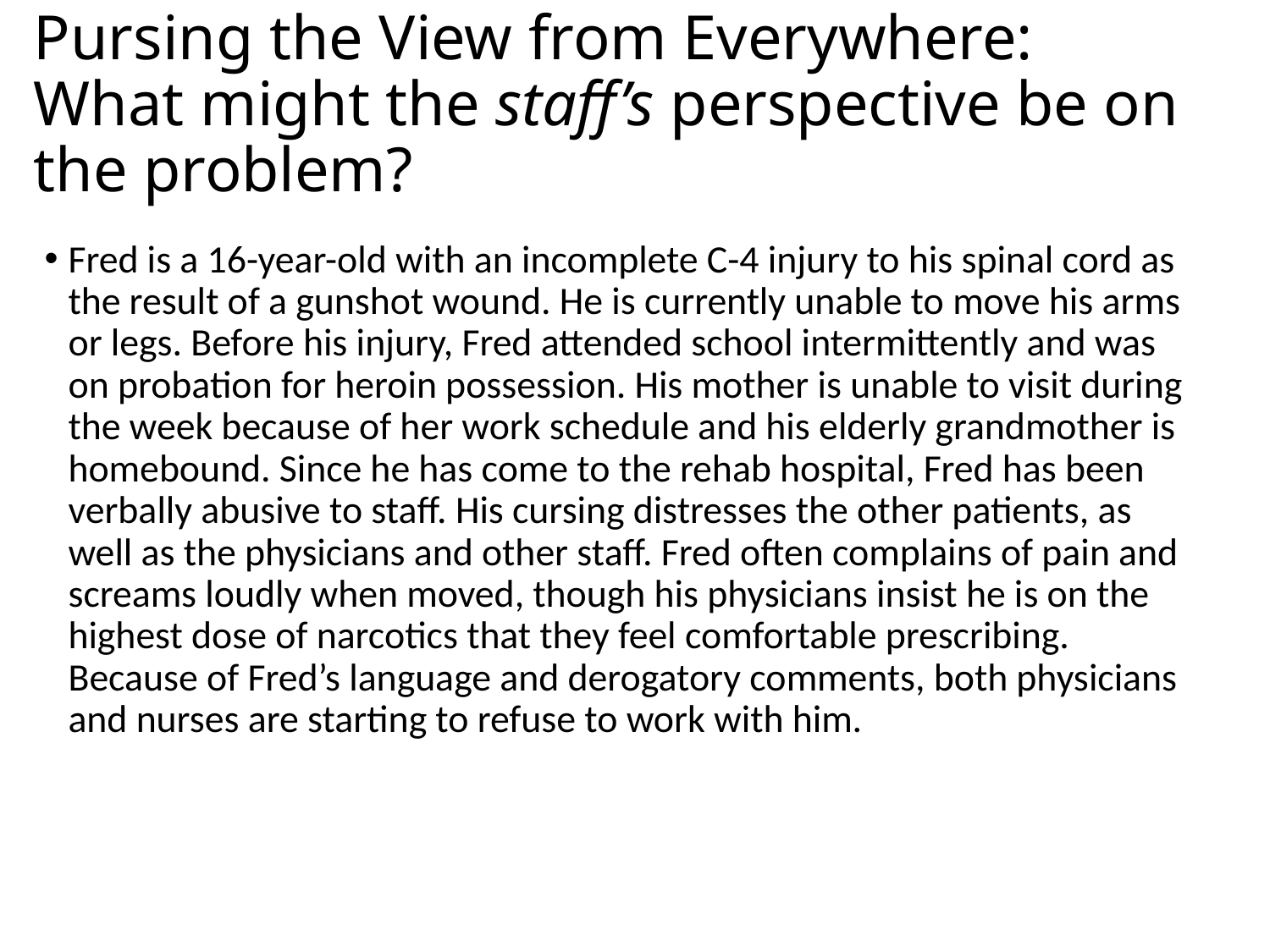

# Pursing the View from Everywhere: What might the staff’s perspective be on the problem?
Fred is a 16-year-old with an incomplete C-4 injury to his spinal cord as the result of a gunshot wound. He is currently unable to move his arms or legs. Before his injury, Fred attended school intermittently and was on probation for heroin possession. His mother is unable to visit during the week because of her work schedule and his elderly grandmother is homebound. Since he has come to the rehab hospital, Fred has been verbally abusive to staff. His cursing distresses the other patients, as well as the physicians and other staff. Fred often complains of pain and screams loudly when moved, though his physicians insist he is on the highest dose of narcotics that they feel comfortable prescribing. Because of Fred’s language and derogatory comments, both physicians and nurses are starting to refuse to work with him.

## Slide 22
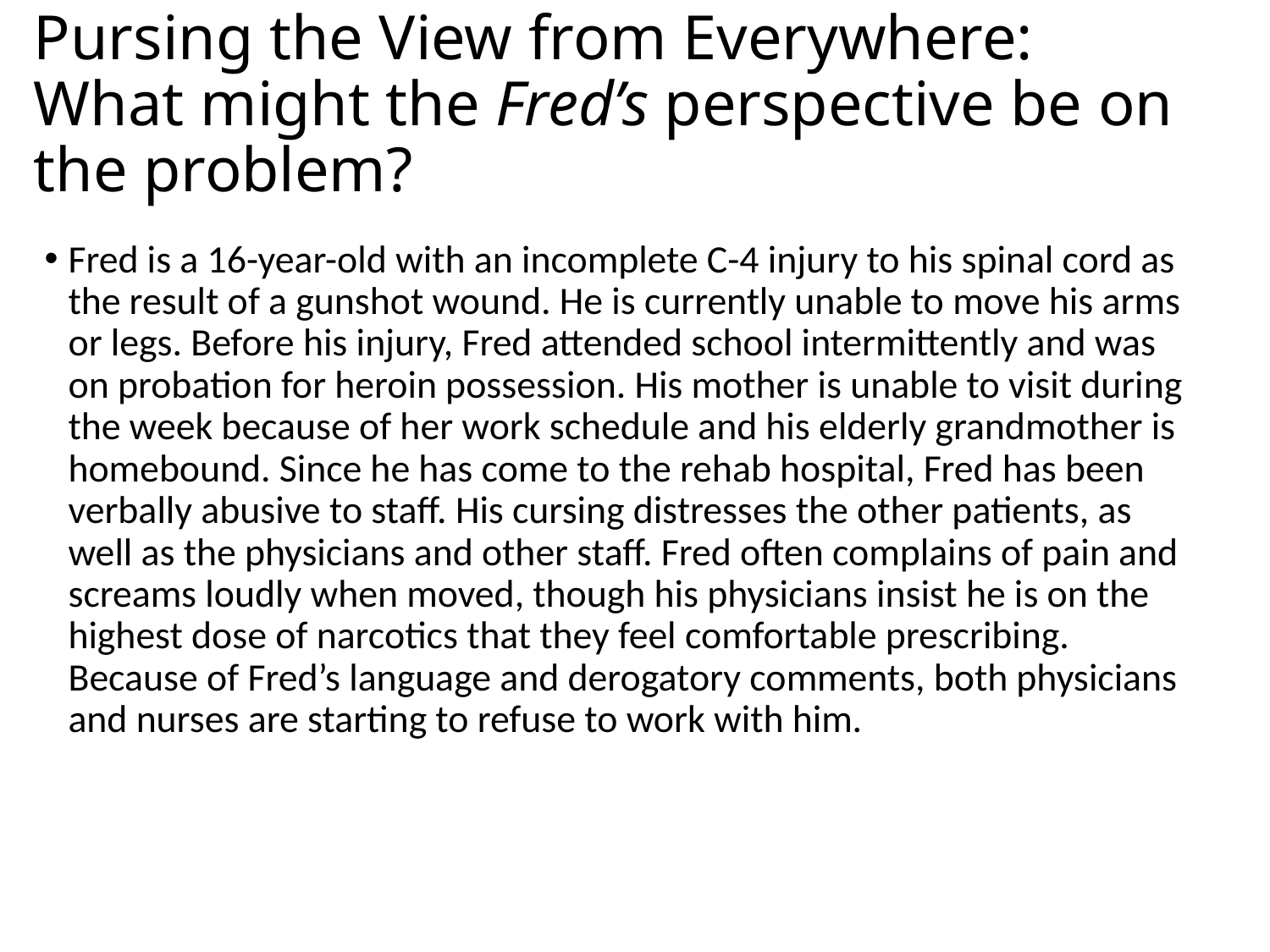

# Pursing the View from Everywhere: What might the Fred’s perspective be on the problem?
Fred is a 16-year-old with an incomplete C-4 injury to his spinal cord as the result of a gunshot wound. He is currently unable to move his arms or legs. Before his injury, Fred attended school intermittently and was on probation for heroin possession. His mother is unable to visit during the week because of her work schedule and his elderly grandmother is homebound. Since he has come to the rehab hospital, Fred has been verbally abusive to staff. His cursing distresses the other patients, as well as the physicians and other staff. Fred often complains of pain and screams loudly when moved, though his physicians insist he is on the highest dose of narcotics that they feel comfortable prescribing. Because of Fred’s language and derogatory comments, both physicians and nurses are starting to refuse to work with him.

## Slide 23
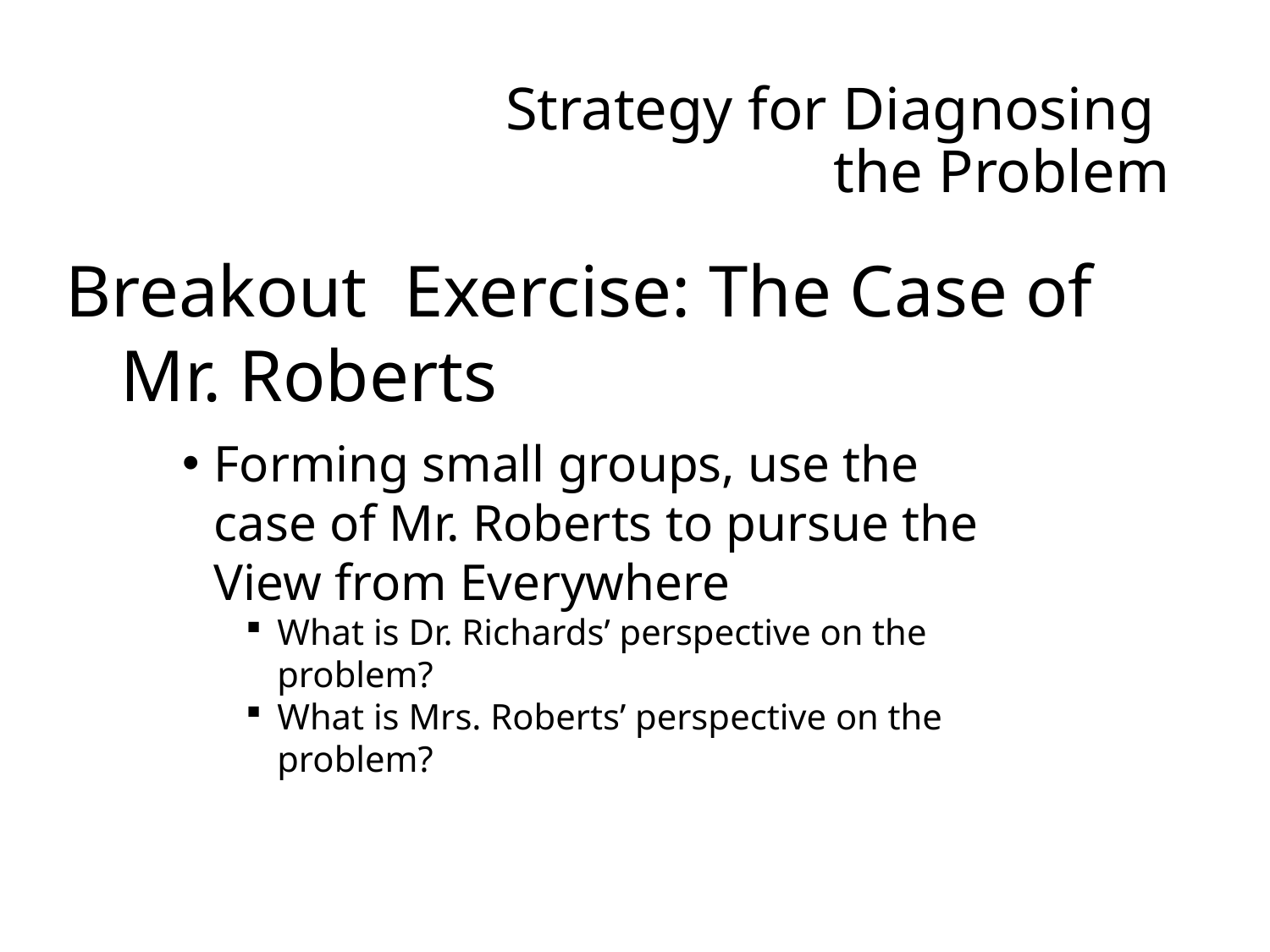

# Strategy for Diagnosing the Problem
Breakout Exercise: The Case of Mr. Roberts
Forming small groups, use the case of Mr. Roberts to pursue the View from Everywhere
What is Dr. Richards’ perspective on the problem?
What is Mrs. Roberts’ perspective on the problem?

## Slide 24
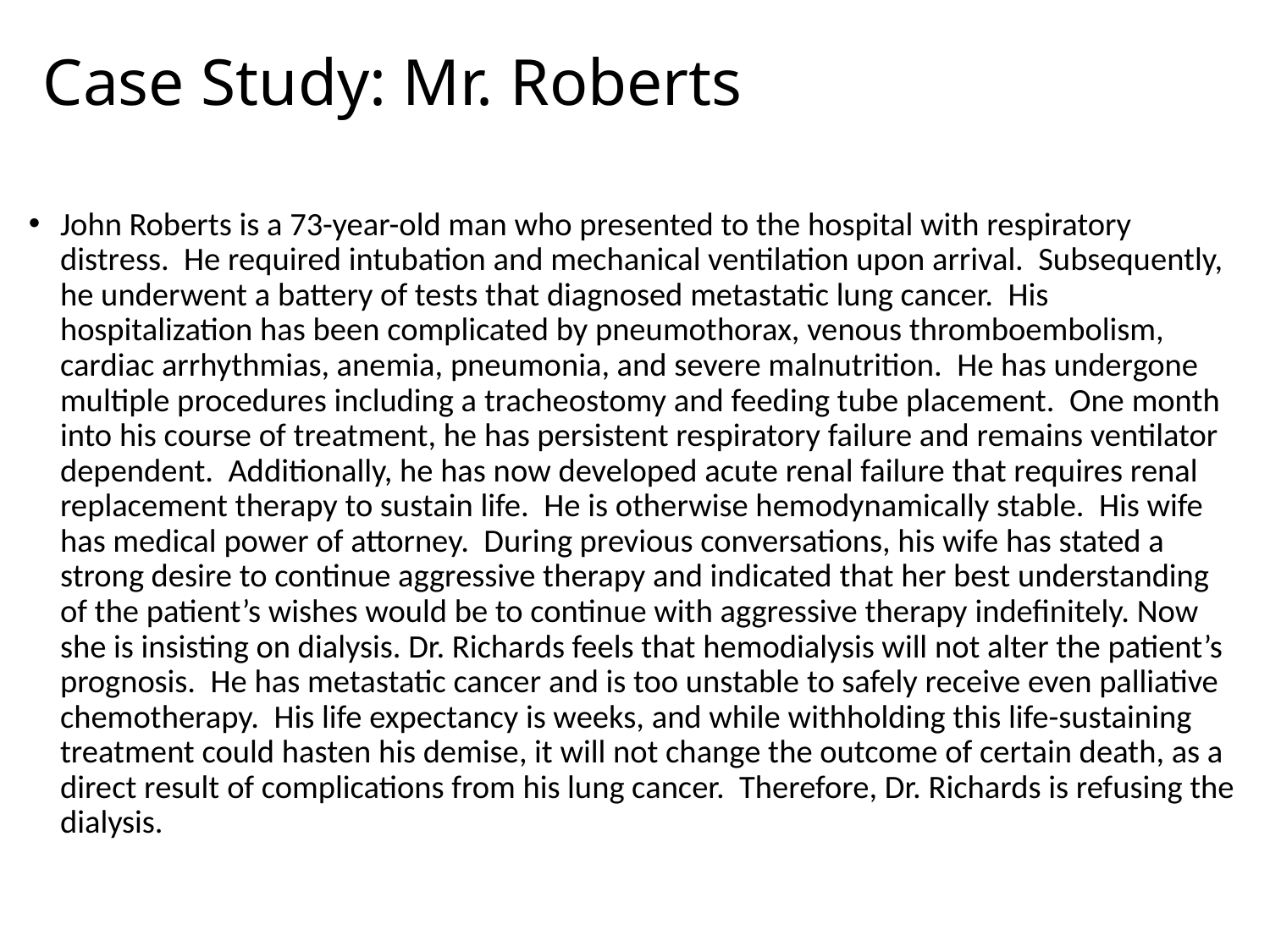

# Case Study: Mr. Roberts
John Roberts is a 73-year-old man who presented to the hospital with respiratory distress. He required intubation and mechanical ventilation upon arrival. Subsequently, he underwent a battery of tests that diagnosed metastatic lung cancer. His hospitalization has been complicated by pneumothorax, venous thromboembolism, cardiac arrhythmias, anemia, pneumonia, and severe malnutrition. He has undergone multiple procedures including a tracheostomy and feeding tube placement. One month into his course of treatment, he has persistent respiratory failure and remains ventilator dependent. Additionally, he has now developed acute renal failure that requires renal replacement therapy to sustain life. He is otherwise hemodynamically stable. His wife has medical power of attorney. During previous conversations, his wife has stated a strong desire to continue aggressive therapy and indicated that her best understanding of the patient’s wishes would be to continue with aggressive therapy indefinitely. Now she is insisting on dialysis. Dr. Richards feels that hemodialysis will not alter the patient’s prognosis. He has metastatic cancer and is too unstable to safely receive even palliative chemotherapy. His life expectancy is weeks, and while withholding this life-sustaining treatment could hasten his demise, it will not change the outcome of certain death, as a direct result of complications from his lung cancer. Therefore, Dr. Richards is refusing the dialysis.

## Slide 25
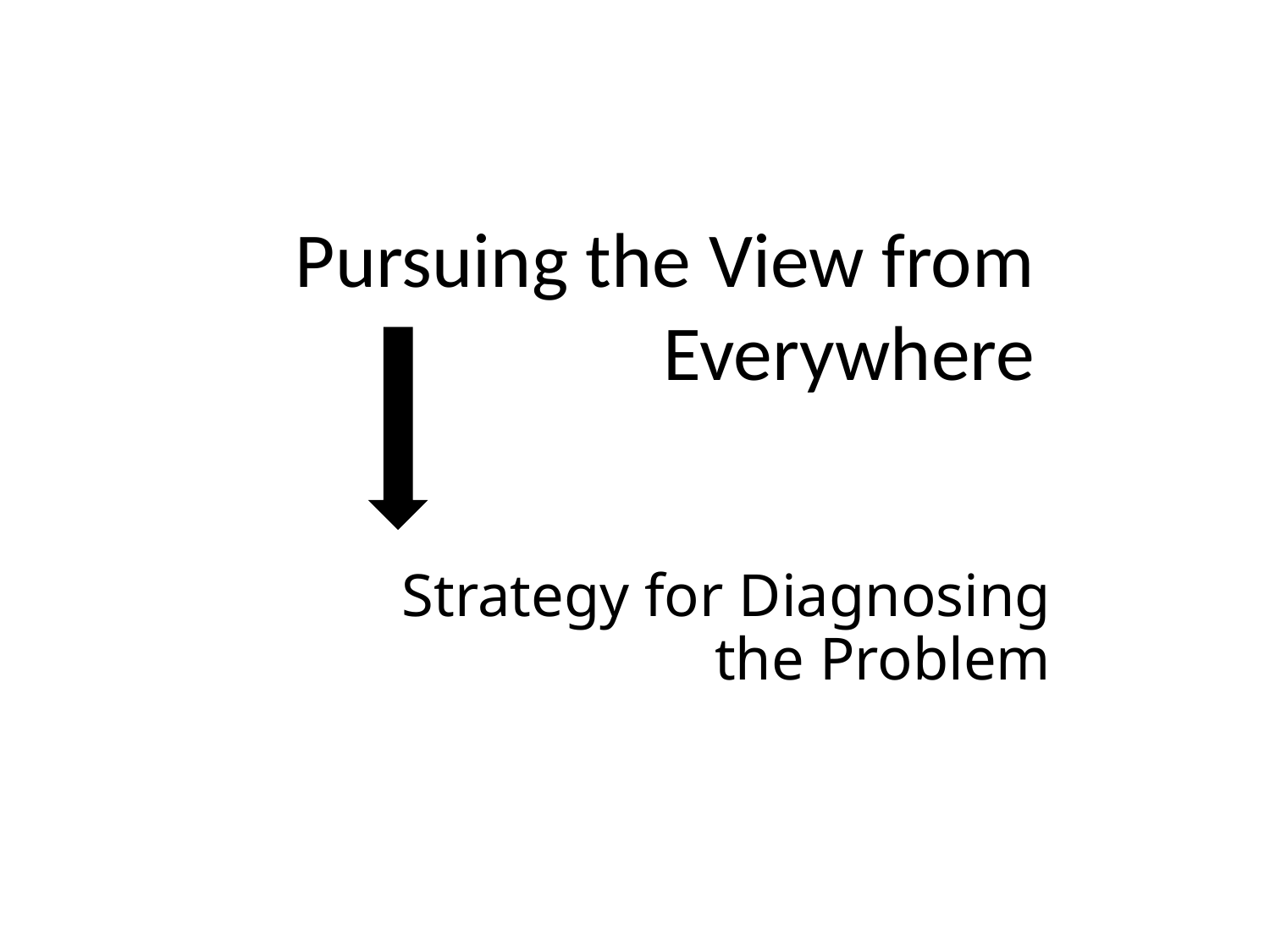

Pursuing the View from Everywhere
# Strategy for Diagnosing the Problem

## Slide 26
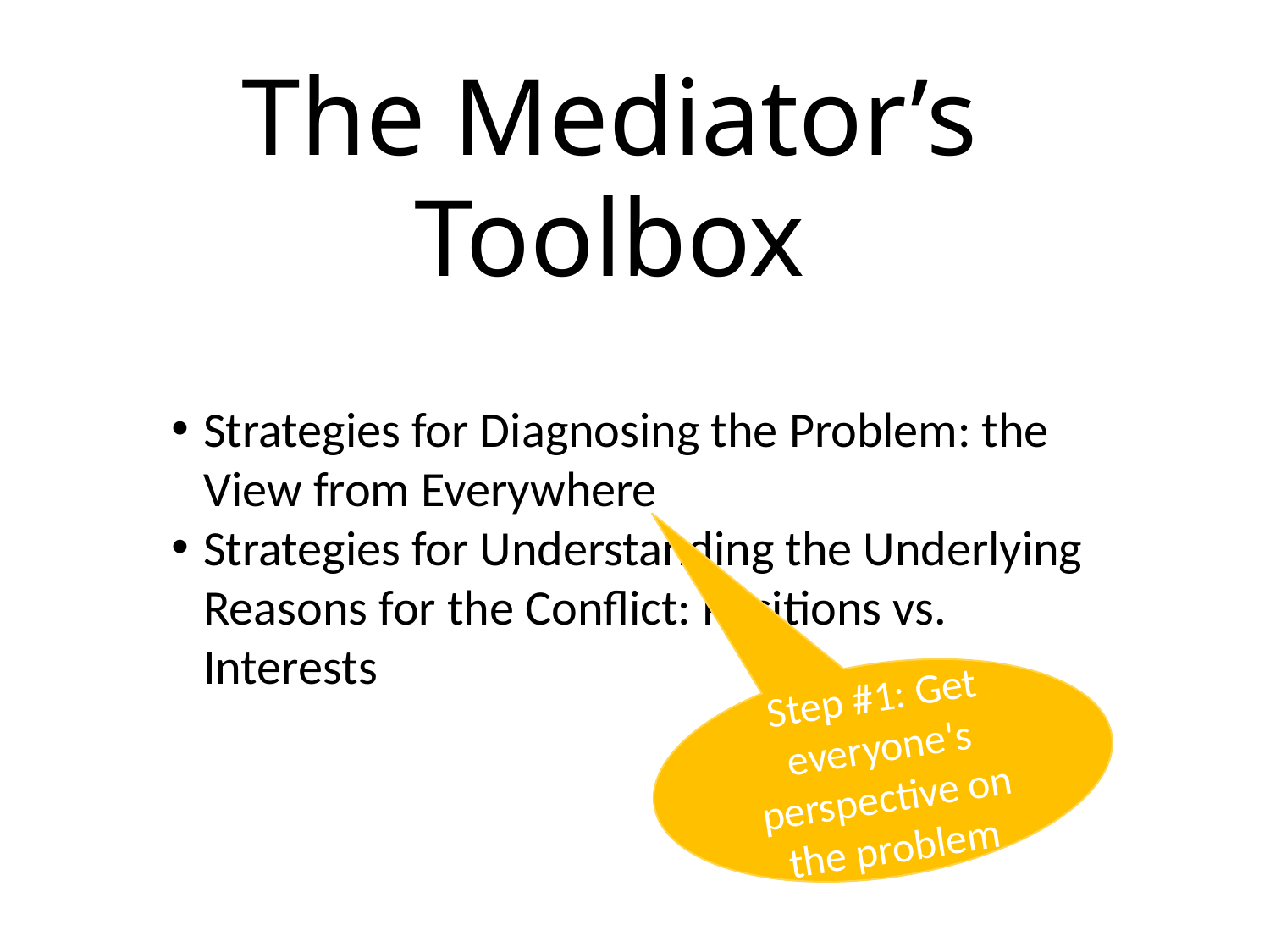

# The Mediator’s Toolbox
Strategies for Diagnosing the Problem: the View from Everywhere
Strategies for Understanding the Underlying Reasons for the Conflict: Positions vs. Interests
Step #1: Get everyone's perspective on the problem

## Slide 27
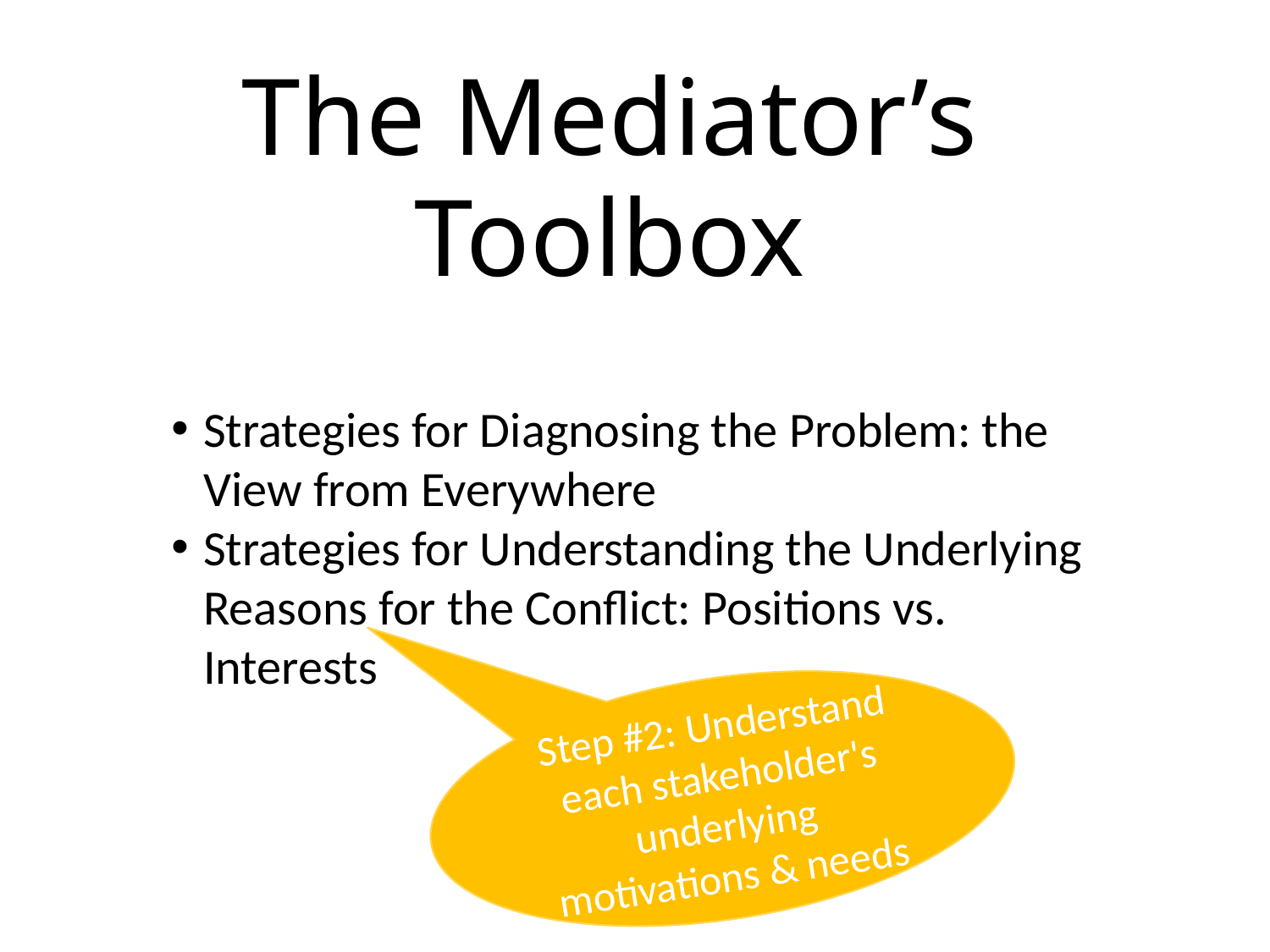

# The Mediator’s Toolbox
Strategies for Diagnosing the Problem: the View from Everywhere
Strategies for Understanding the Underlying Reasons for the Conflict: Positions vs. Interests
Step #2: Understand each stakeholder's underlying motivations & needs

## Slide 28
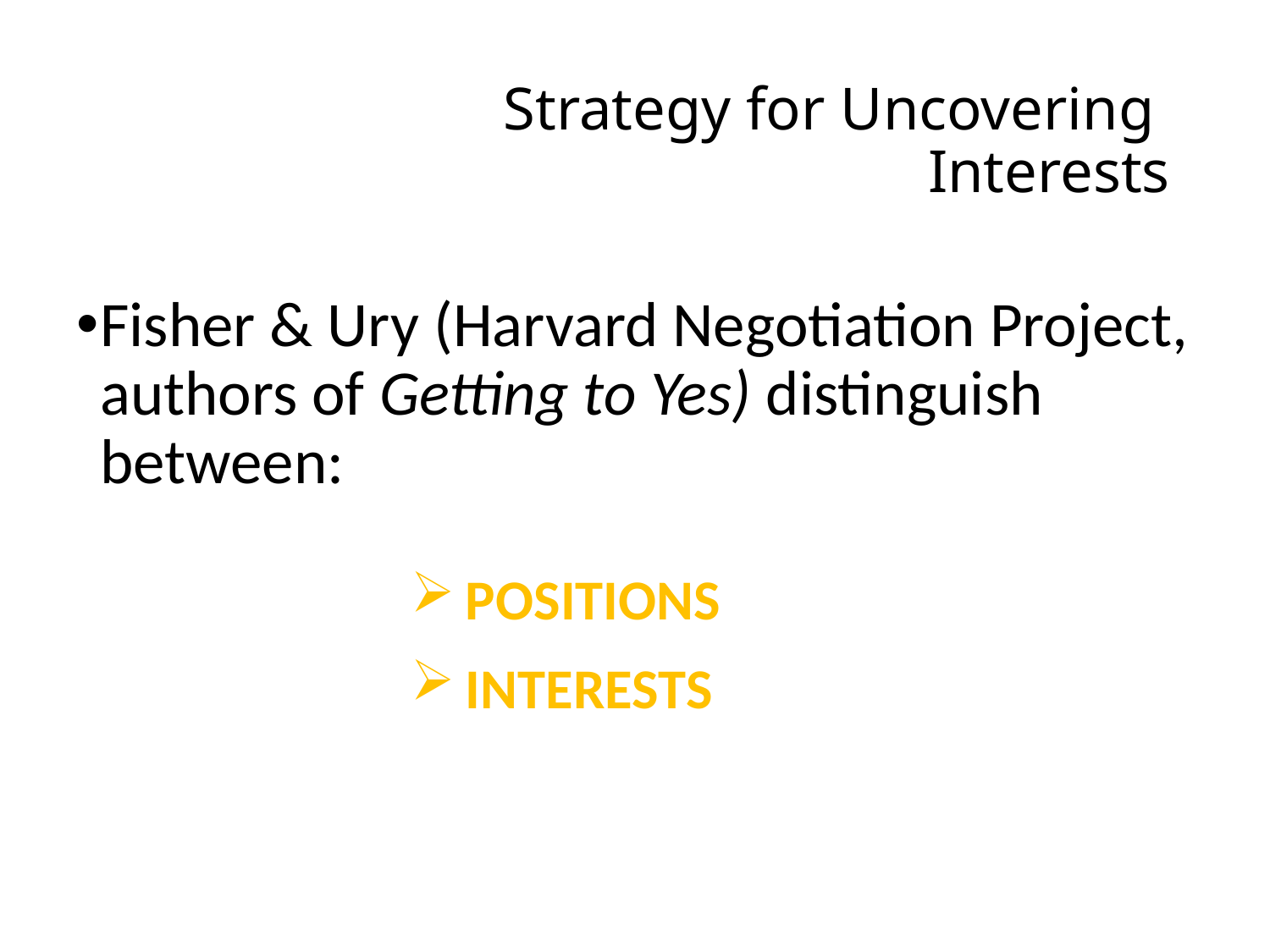

# Strategy for Uncovering Interests
Fisher & Ury (Harvard Negotiation Project, authors of Getting to Yes) distinguish between:
POSITIONS
INTERESTS

## Slide 29
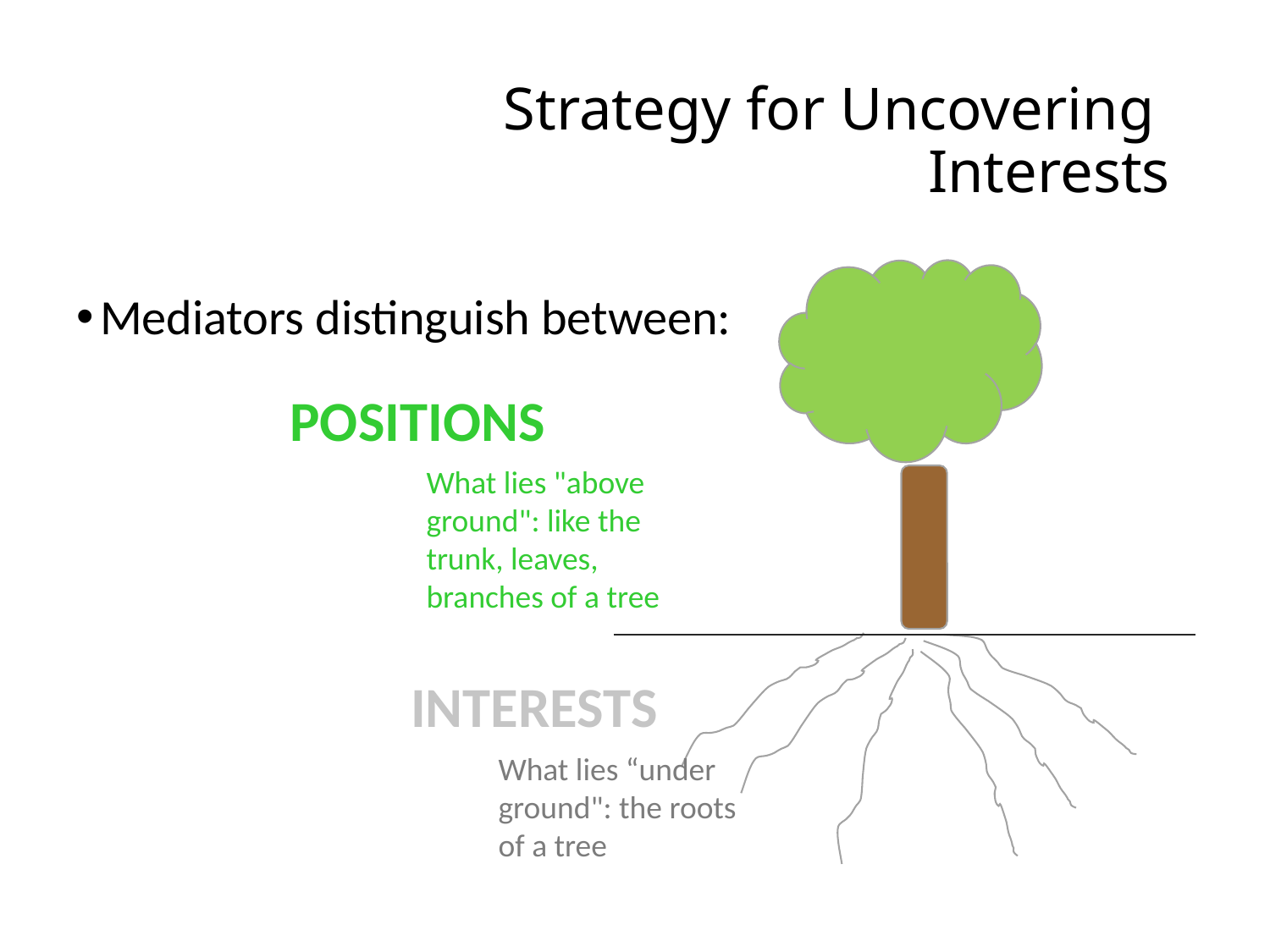

# Strategy for Uncovering Interests
Mediators distinguish between:
POSITIONS
INTERESTS
What lies "above ground": like the trunk, leaves, branches of a tree
What lies “under ground": the roots of a tree

## Slide 30
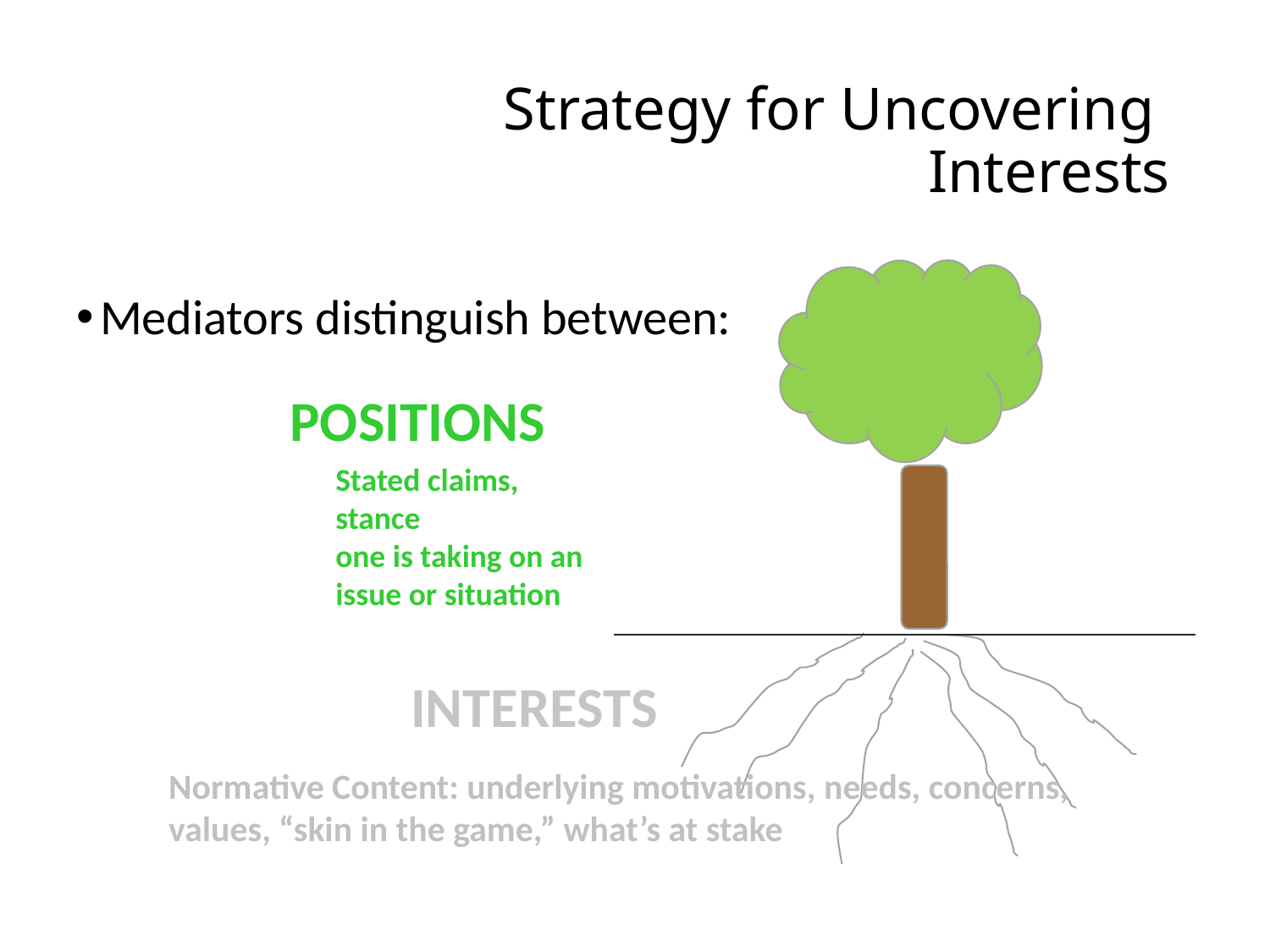

# Strategy for Uncovering Interests
Mediators distinguish between:
POSITIONS
INTERESTS
Stated claims, stance
one is taking on an issue or situation
Normative Content: underlying motivations, needs, concerns, values, “skin in the game,” what’s at stake

## Slide 31
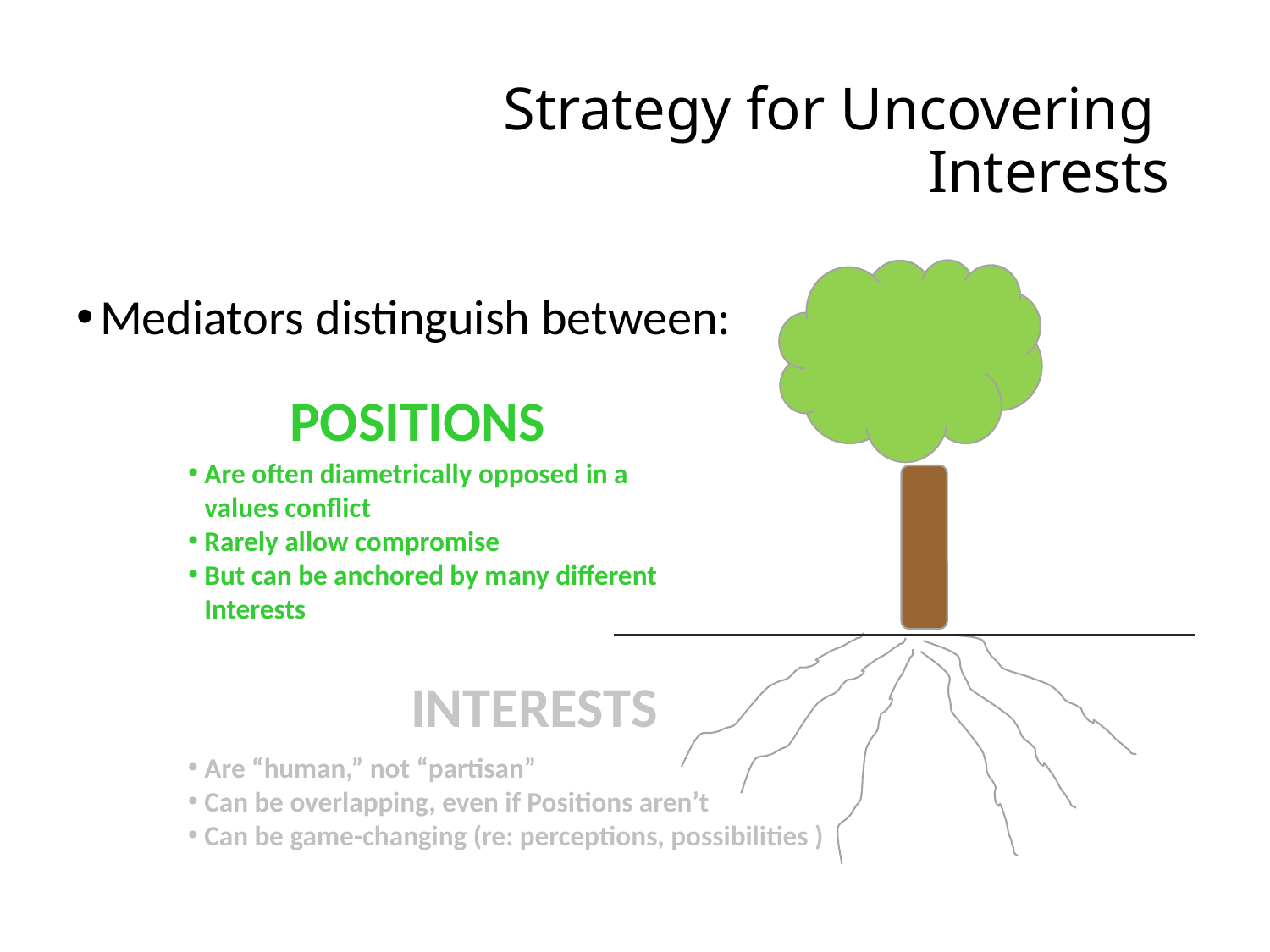

# Strategy for Uncovering Interests
Mediators distinguish between:
POSITIONS
INTERESTS
Are often diametrically opposed in a values conflict
Rarely allow compromise
But can be anchored by many different Interests
Are “human,” not “partisan”
Can be overlapping, even if Positions aren’t
Can be game-changing (re: perceptions, possibilities )

## Slide 32
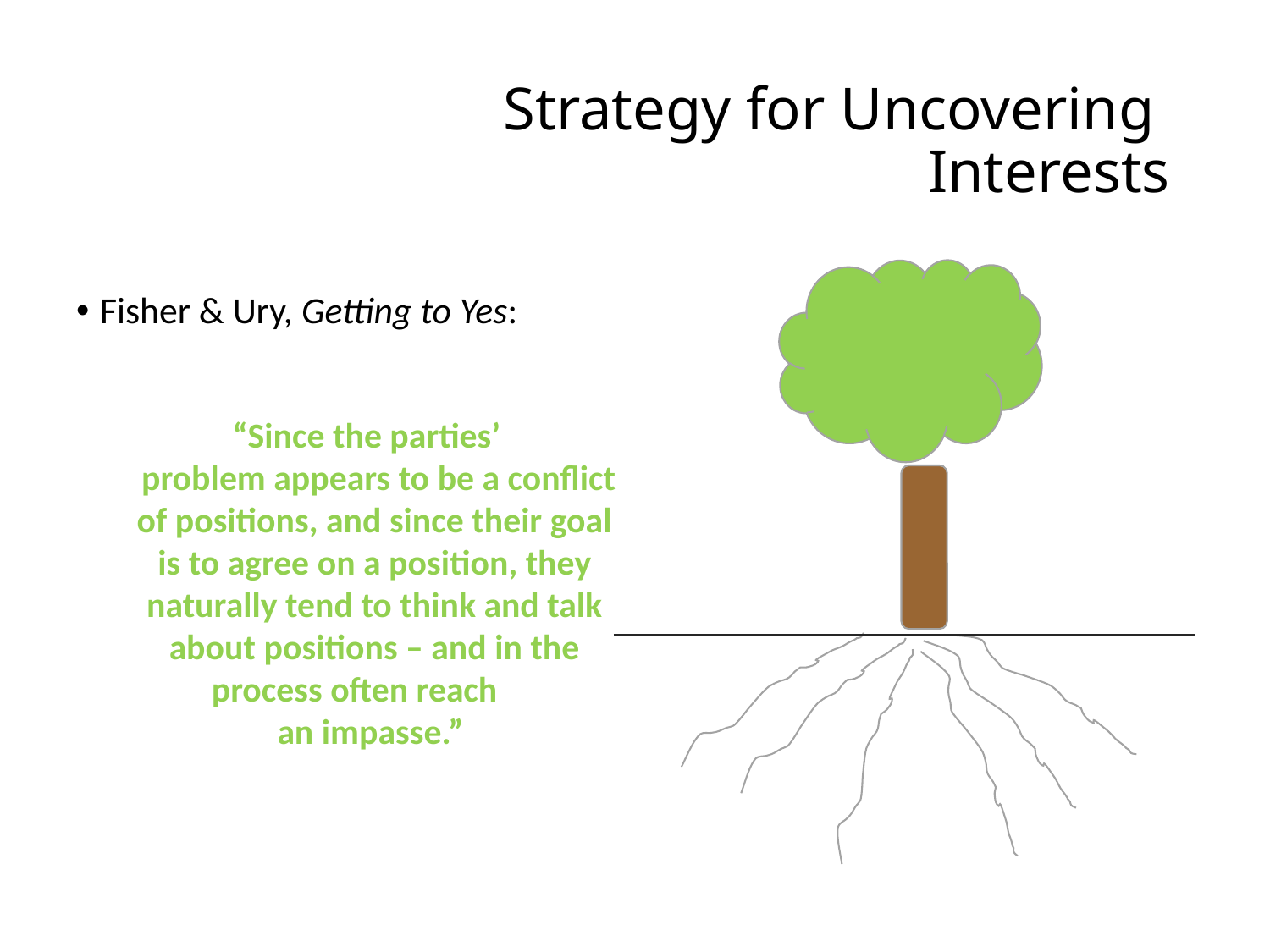

# Strategy for Uncovering Interests
Fisher & Ury, Getting to Yes:
“Since the parties’
 problem appears to be a conflict of positions, and since their goal is to agree on a position, they naturally tend to think and talk about positions – and in the process often reach
an impasse.”

## Slide 33
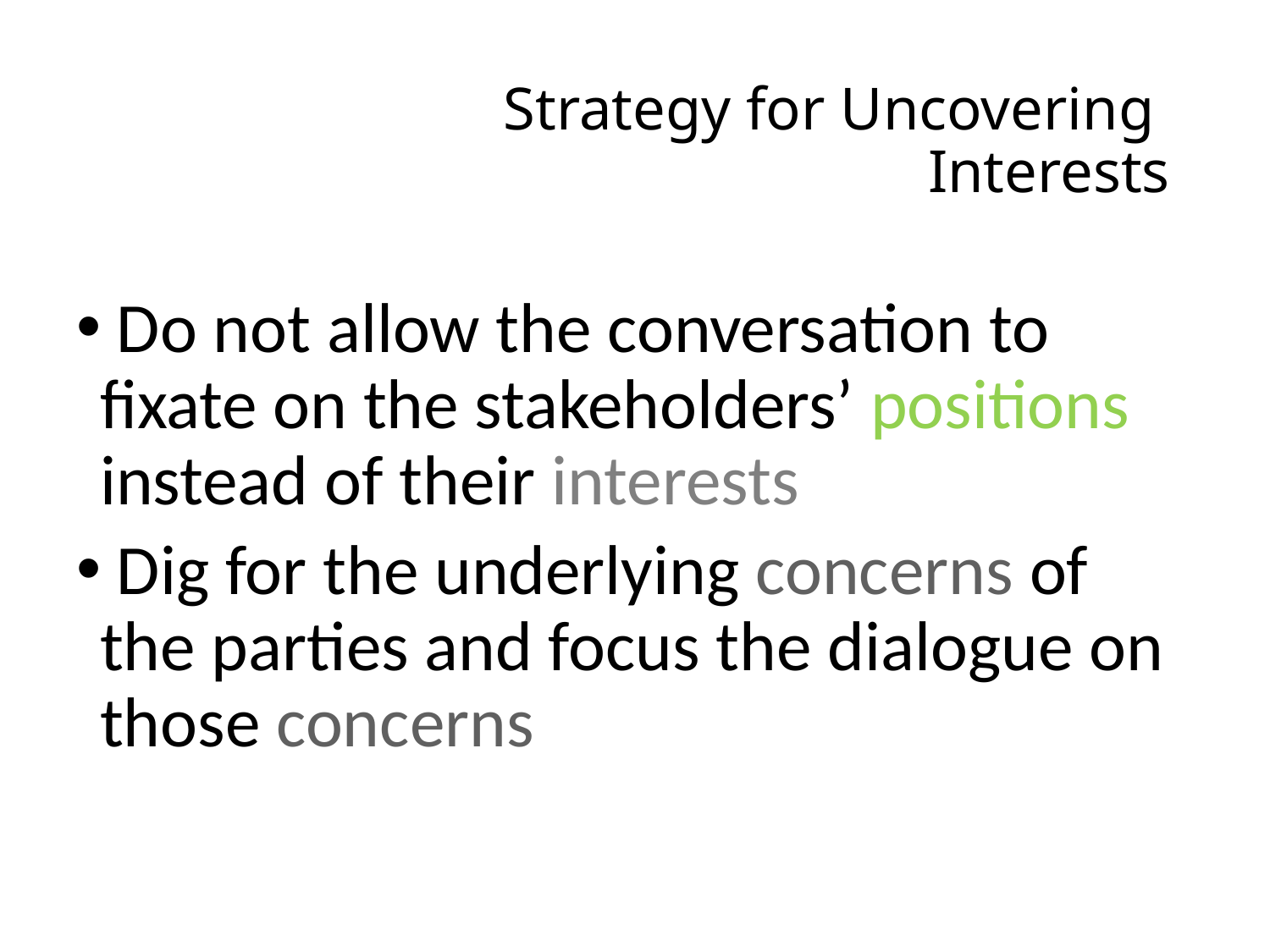

# Strategy for Uncovering Interests
 Do not allow the conversation to fixate on the stakeholders’ positions instead of their interests
 Dig for the underlying concerns of the parties and focus the dialogue on those concerns

## Slide 34
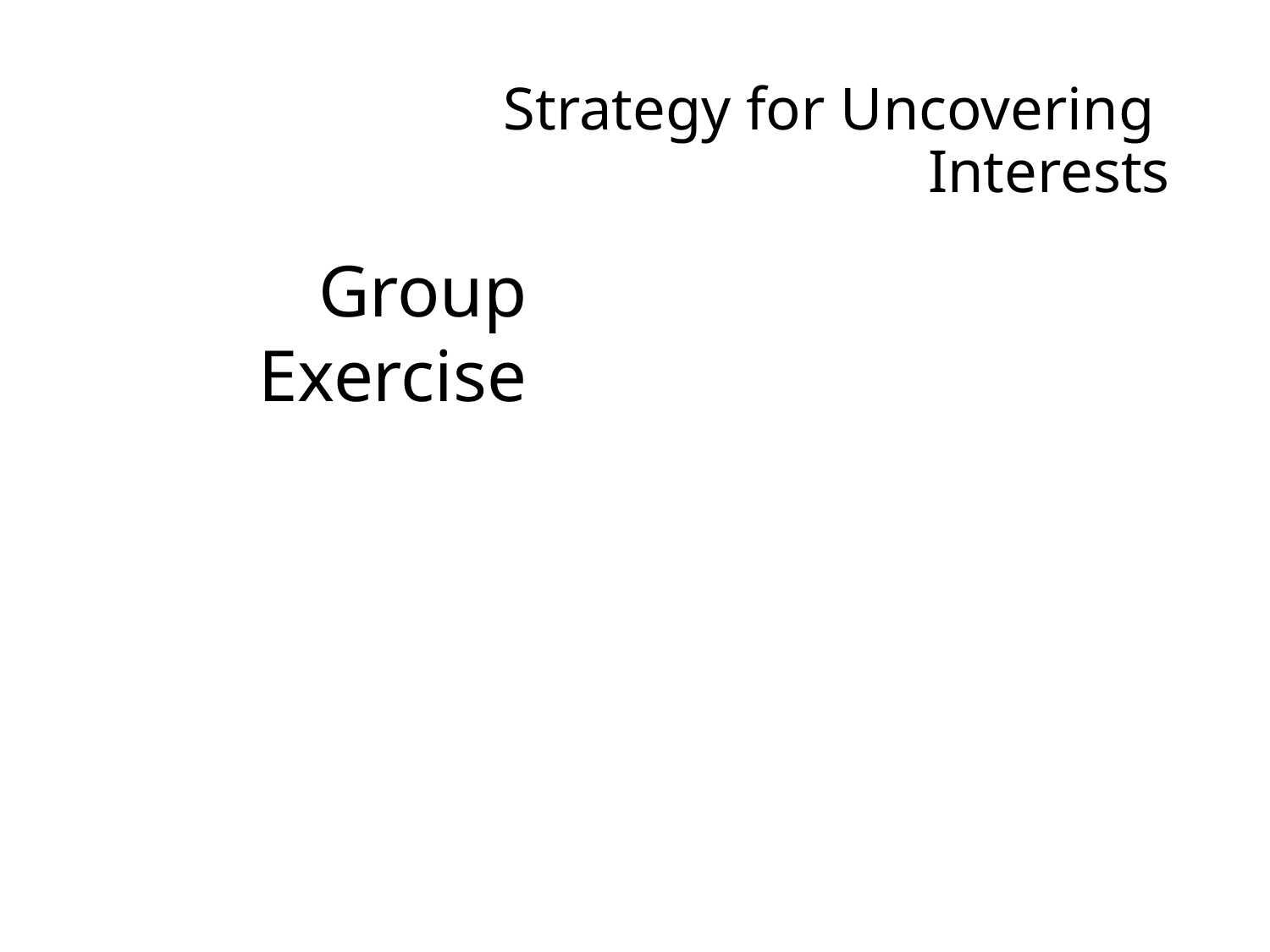

# Strategy for Uncovering Interests
Group Exercise

## Slide 35
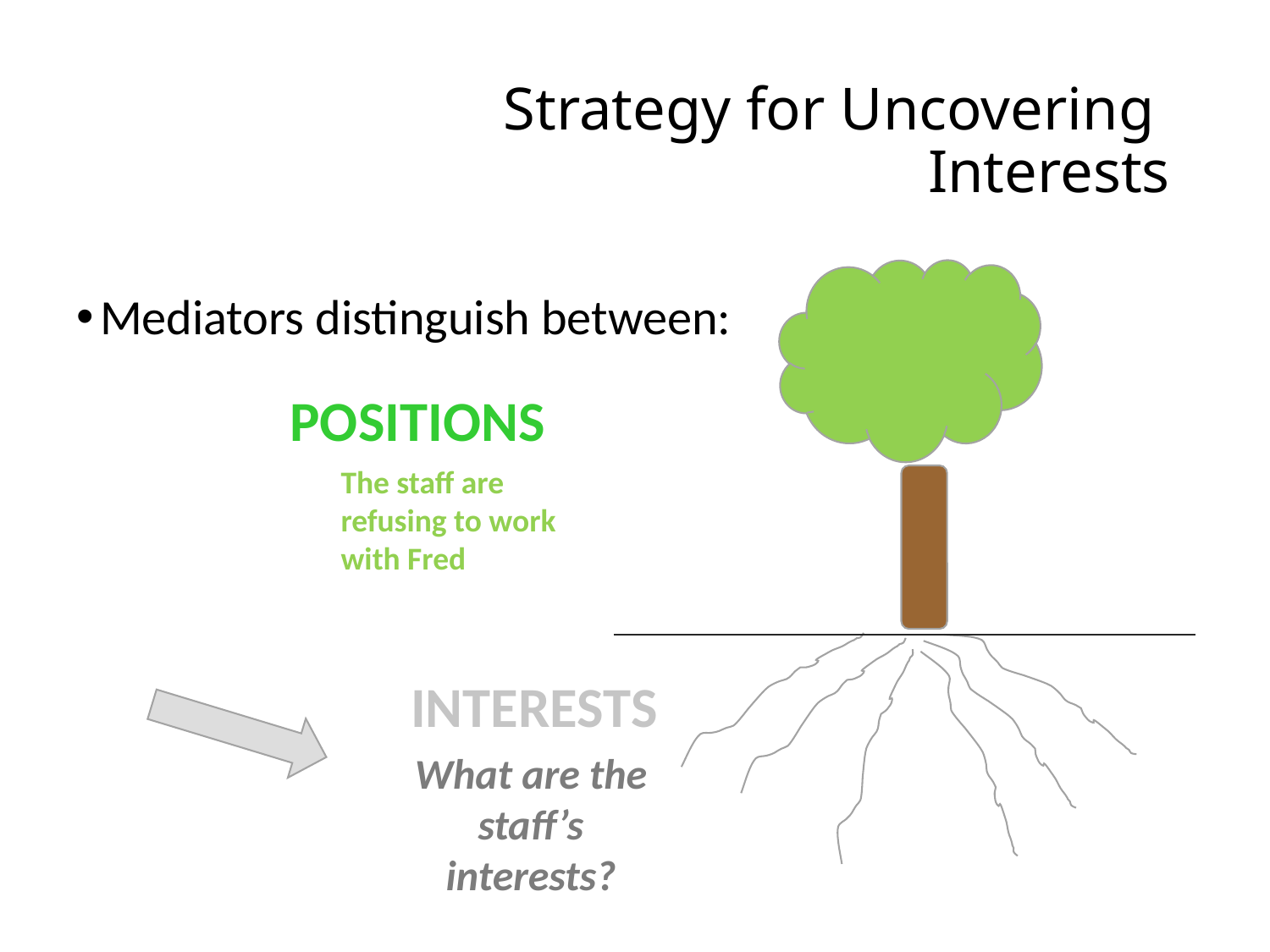

# Strategy for Uncovering Interests
Mediators distinguish between:
POSITIONS
INTERESTS
The staff are refusing to work with Fred
What are the staff’s interests?

## Slide 36
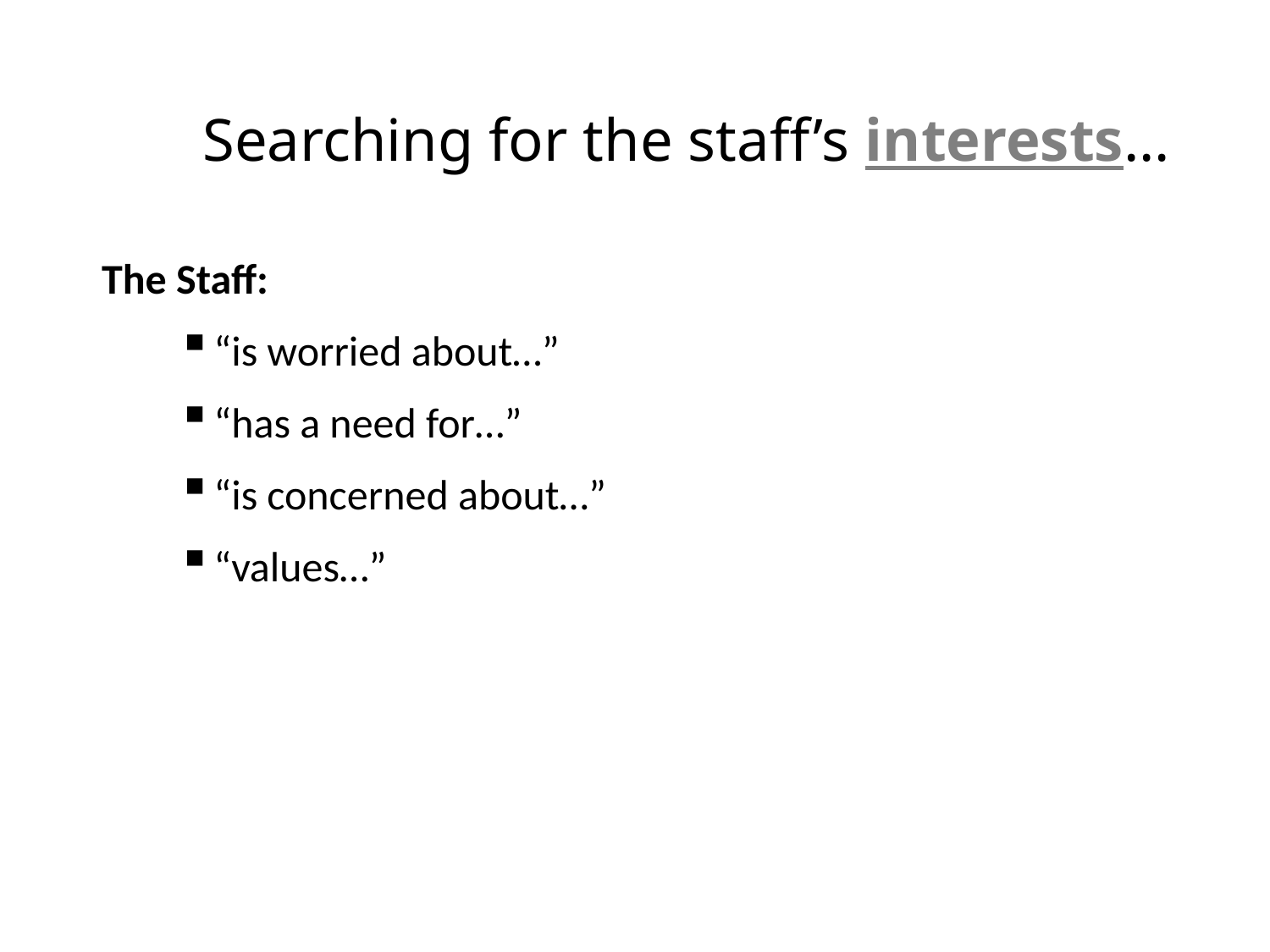

# Searching for the staff’s interests…
The Staff:
“is worried about…”
“has a need for…”
“is concerned about…”
“values…”

## Slide 37
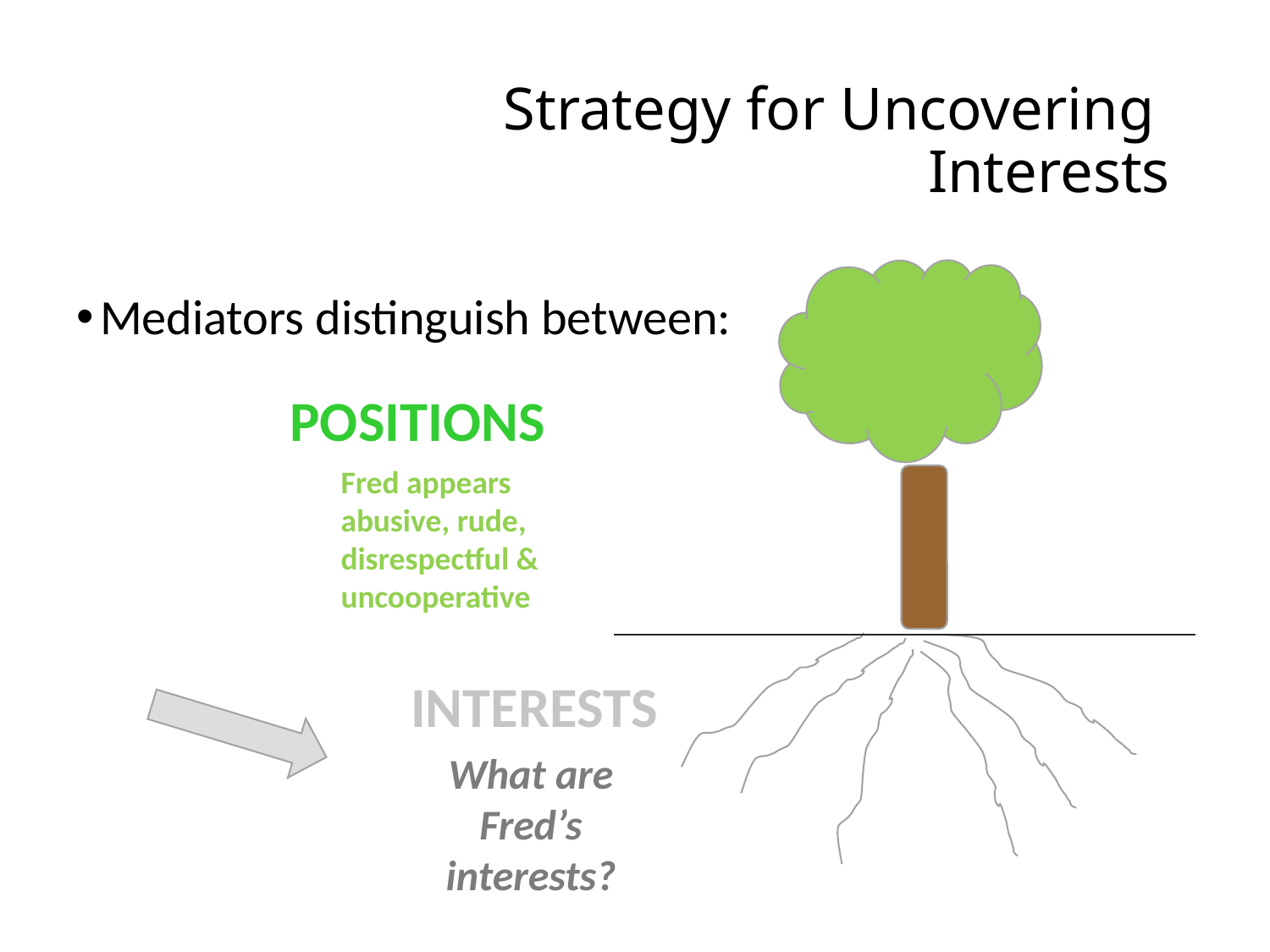

# Strategy for Uncovering Interests
Mediators distinguish between:
POSITIONS
INTERESTS
Fred appears abusive, rude, disrespectful & uncooperative
What are Fred’s interests?

## Slide 38
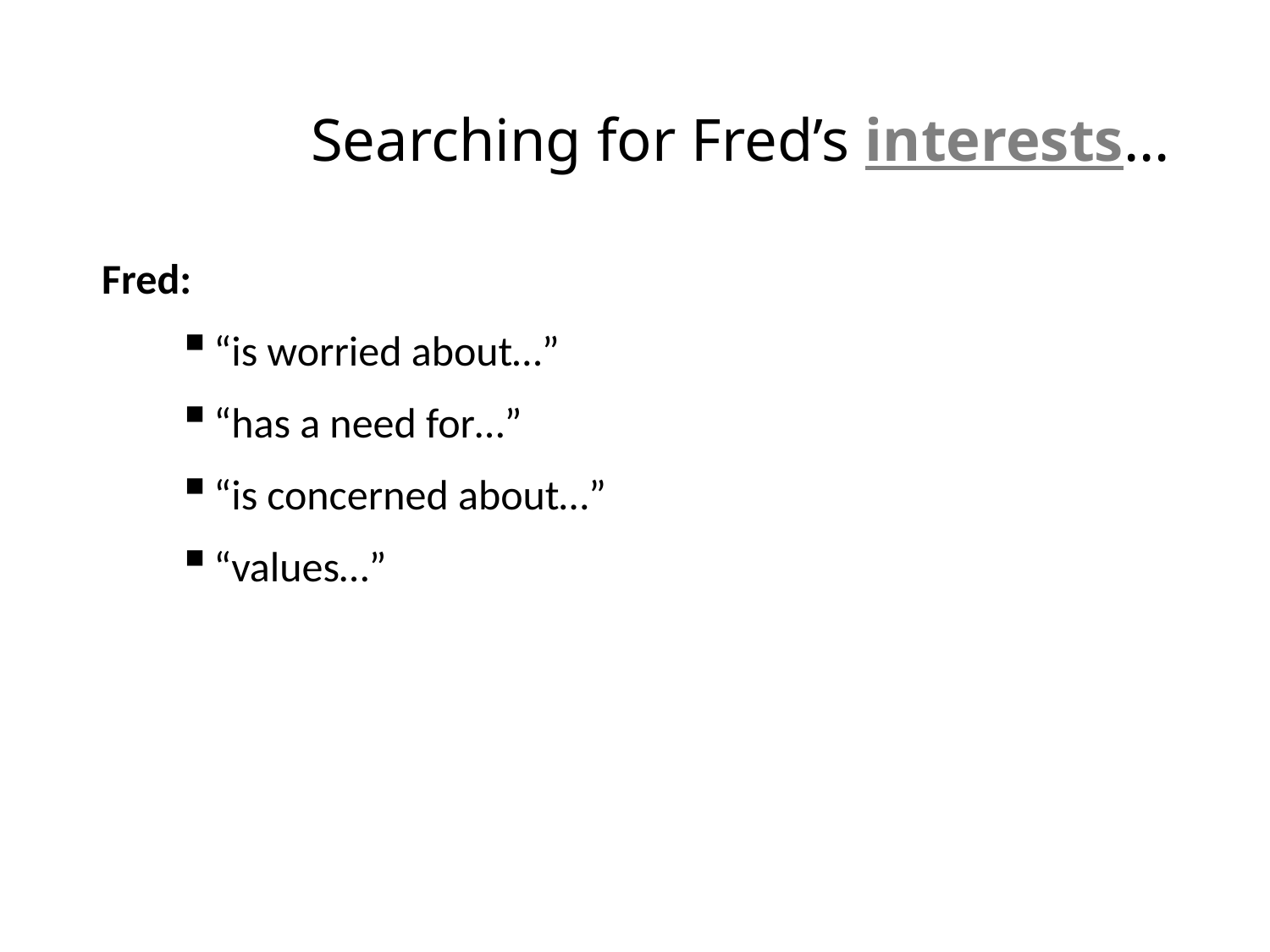

# Searching for Fred’s interests…
Fred:
“is worried about…”
“has a need for…”
“is concerned about…”
“values…”

## Slide 39
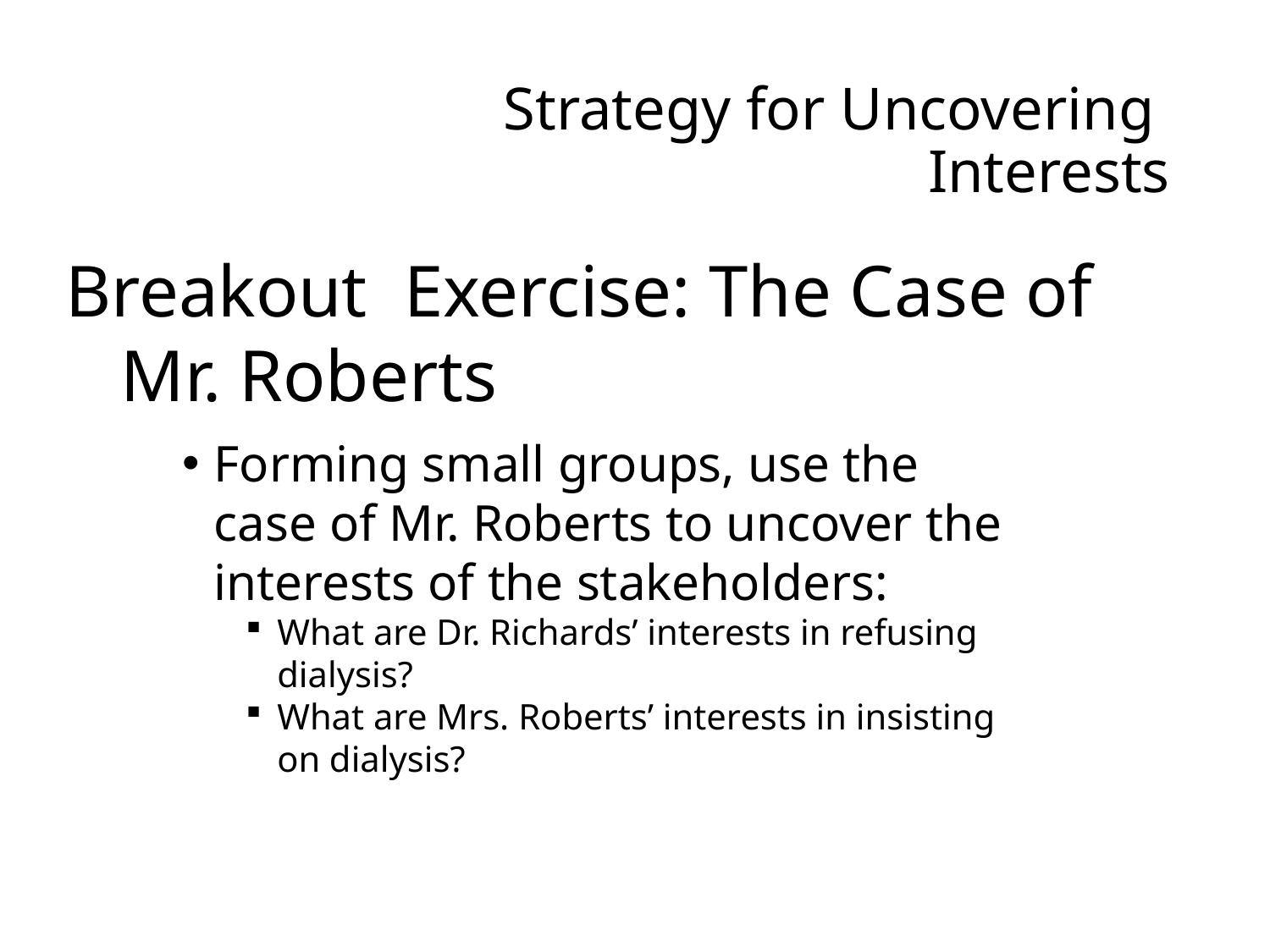

# Strategy for Uncovering Interests
Breakout Exercise: The Case of Mr. Roberts
Forming small groups, use the case of Mr. Roberts to uncover the interests of the stakeholders:
What are Dr. Richards’ interests in refusing dialysis?
What are Mrs. Roberts’ interests in insisting on dialysis?

## Slide 40
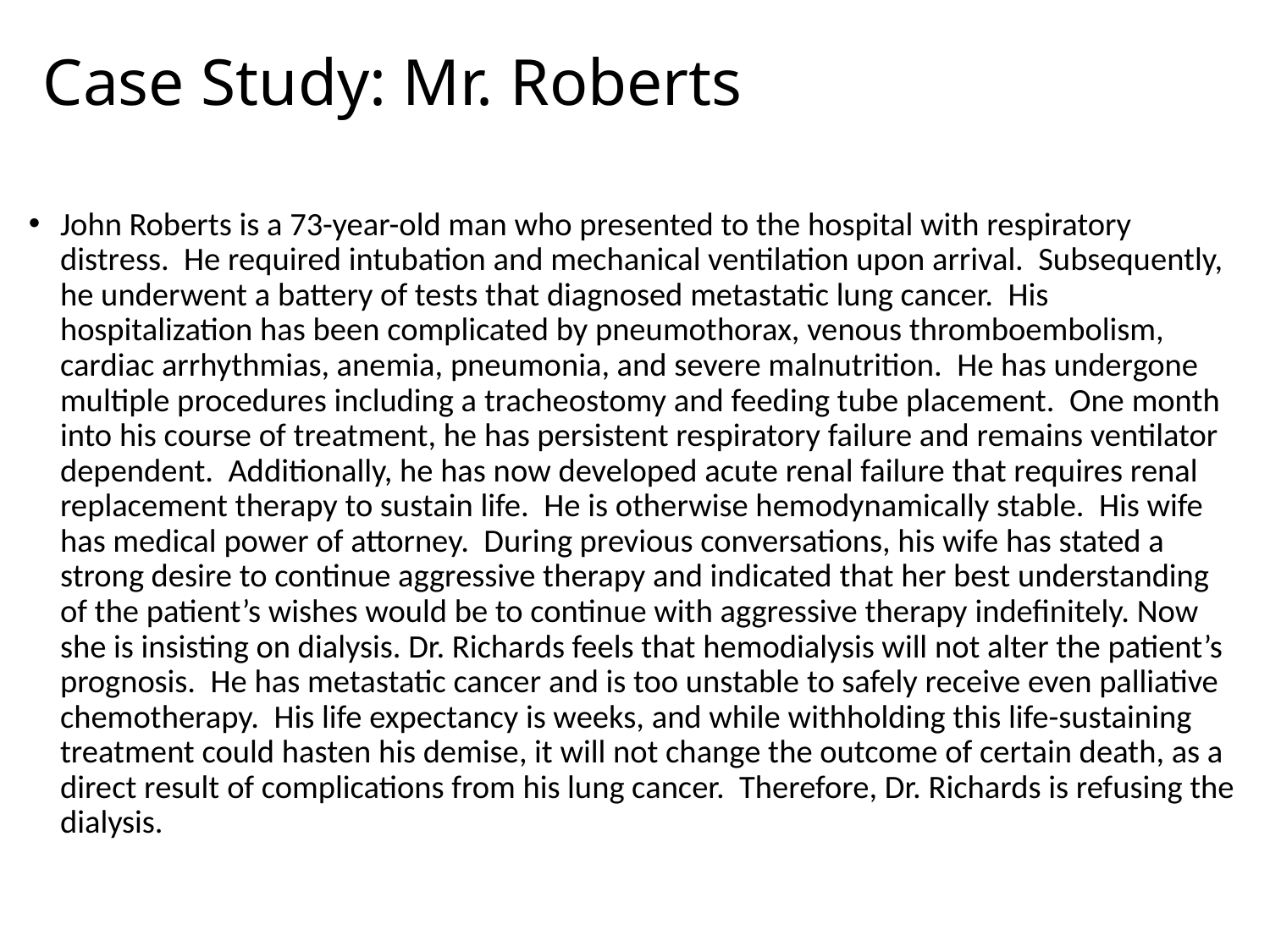

# Case Study: Mr. Roberts
John Roberts is a 73-year-old man who presented to the hospital with respiratory distress. He required intubation and mechanical ventilation upon arrival. Subsequently, he underwent a battery of tests that diagnosed metastatic lung cancer. His hospitalization has been complicated by pneumothorax, venous thromboembolism, cardiac arrhythmias, anemia, pneumonia, and severe malnutrition. He has undergone multiple procedures including a tracheostomy and feeding tube placement. One month into his course of treatment, he has persistent respiratory failure and remains ventilator dependent. Additionally, he has now developed acute renal failure that requires renal replacement therapy to sustain life. He is otherwise hemodynamically stable. His wife has medical power of attorney. During previous conversations, his wife has stated a strong desire to continue aggressive therapy and indicated that her best understanding of the patient’s wishes would be to continue with aggressive therapy indefinitely. Now she is insisting on dialysis. Dr. Richards feels that hemodialysis will not alter the patient’s prognosis. He has metastatic cancer and is too unstable to safely receive even palliative chemotherapy. His life expectancy is weeks, and while withholding this life-sustaining treatment could hasten his demise, it will not change the outcome of certain death, as a direct result of complications from his lung cancer. Therefore, Dr. Richards is refusing the dialysis.

## Slide 41
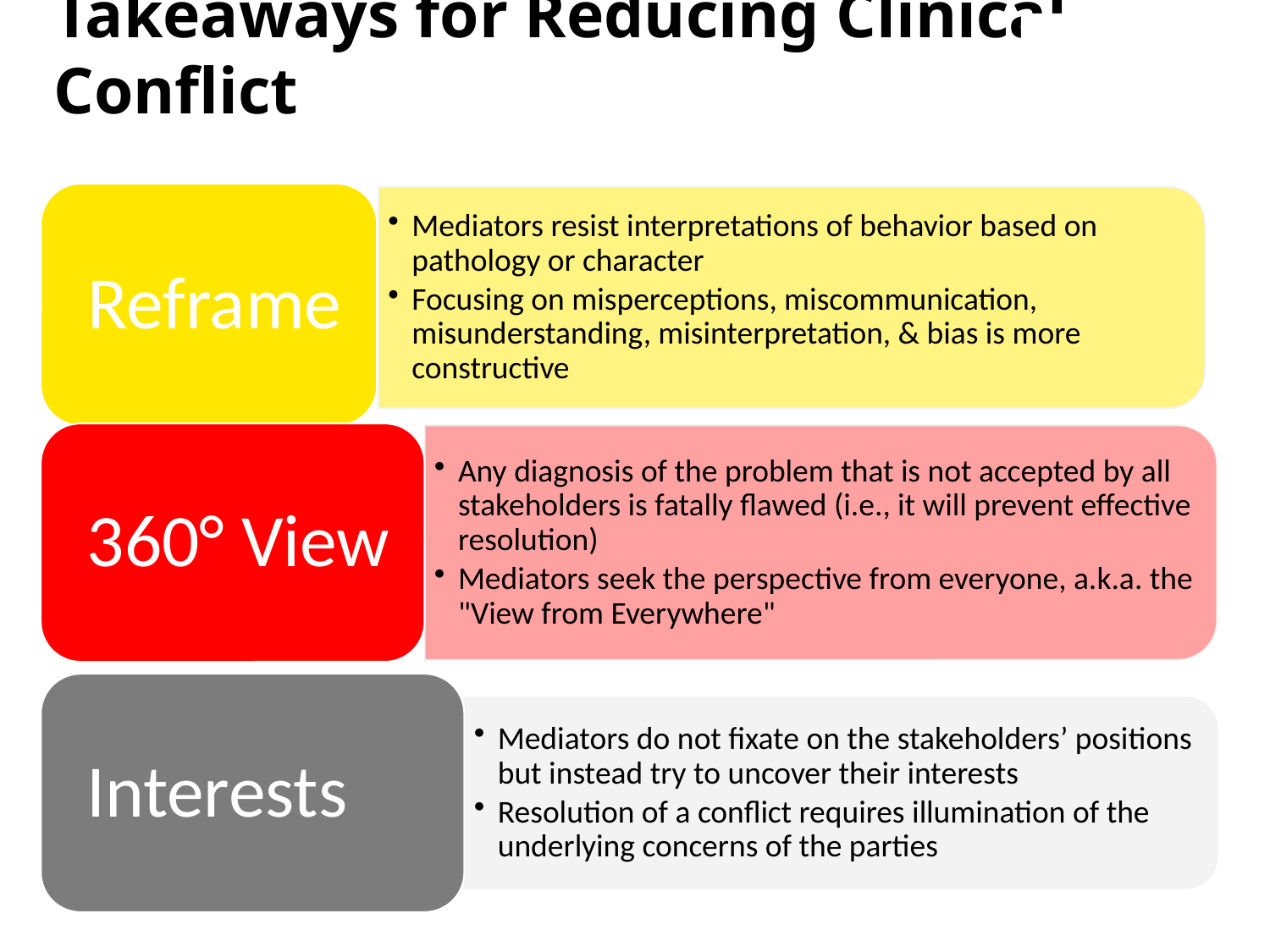

# Takeaways for Reducing Clinical Conflict
Myanmar Identity

## Slide 42
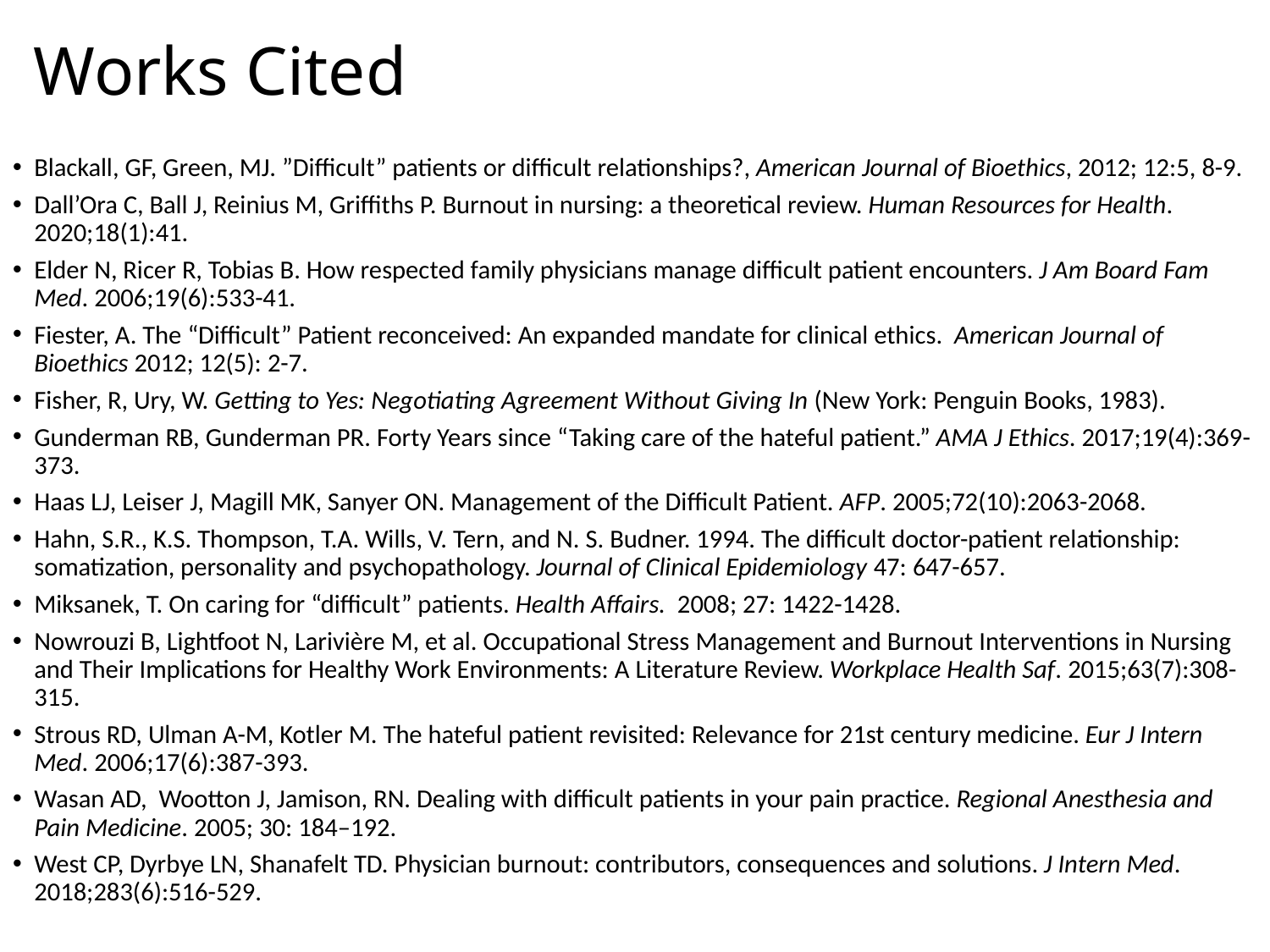

# Works Cited
Blackall, GF, Green, MJ. ”Difficult” patients or difficult relationships?, American Journal of Bioethics, 2012; 12:5, 8-9.
Dall’Ora C, Ball J, Reinius M, Griffiths P. Burnout in nursing: a theoretical review. Human Resources for Health. 2020;18(1):41.
Elder N, Ricer R, Tobias B. How respected family physicians manage difficult patient encounters. J Am Board Fam Med. 2006;19(6):533-41.
Fiester, A. The “Difficult” Patient reconceived: An expanded mandate for clinical ethics. American Journal of Bioethics 2012; 12(5): 2-7.
Fisher, R, Ury, W. Getting to Yes: Negotiating Agreement Without Giving In (New York: Penguin Books, 1983).
Gunderman RB, Gunderman PR. Forty Years since “Taking care of the hateful patient.” AMA J Ethics. 2017;19(4):369-373.
Haas LJ, Leiser J, Magill MK, Sanyer ON. Management of the Difficult Patient. AFP. 2005;72(10):2063-2068.
Hahn, S.R., K.S. Thompson, T.A. Wills, V. Tern, and N. S. Budner. 1994. The difficult doctor-patient relationship: somatization, personality and psychopathology. Journal of Clinical Epidemiology 47: 647-657.
Miksanek, T. On caring for “difficult” patients. Health Affairs. 2008; 27: 1422-1428.
Nowrouzi B, Lightfoot N, Larivière M, et al. Occupational Stress Management and Burnout Interventions in Nursing and Their Implications for Healthy Work Environments: A Literature Review. Workplace Health Saf. 2015;63(7):308-315.
Strous RD, Ulman A-M, Kotler M. The hateful patient revisited: Relevance for 21st century medicine. Eur J Intern Med. 2006;17(6):387-393.
Wasan AD, Wootton J, Jamison, RN. Dealing with difficult patients in your pain practice. Regional Anesthesia and Pain Medicine. 2005; 30: 184–192.
West CP, Dyrbye LN, Shanafelt TD. Physician burnout: contributors, consequences and solutions. J Intern Med. 2018;283(6):516-529.
