# Supplementary material for: Using a Mediator's Toolbox: Reducing Clinical Conflict by Learning to Reconceive the “Difficult” Patient or Family
Source: MedEdPORTAL. 2023 Jul 14;19:11324. doi: 10.15766/mep_2374-8265.11324 (PMC10345165; doi:10.15766/mep_2374-8265.11324)
Supplement: Supplementary file 1 — Using the Mediators Toolbox Presentation.pptxView From Everywhere Case Study.docxPositions vs. Interests Case Study.docxWorkshop Evaluation.docx [file mep_2374-8265.11324-s001.zip › B. View From Everywhere Case Study.docx]

**Appendix B: Using the Mediator’s Toolbox**

**“View From Everywhere” Breakout Session**

**Case Study: Mr. Roberts**

**Case:**

**John Roberts is a 73-year-old man who presented to the hospital with respiratory distress. He required intubation and mechanical ventilation upon arrival. Subsequently, he underwent a battery of tests that diagnosed metastatic lung cancer. His hospitalization has been complicated by pneumothorax, venous thromboembolism, cardiac arrhythmias, anemia, pneumonia, and severe malnutrition. He has undergone multiple procedures including a tracheostomy and feeding tube placement. One month into his course of treatment, he has persistent respiratory failure and remains ventilator dependent. Additionally, he has now developed acute renal failure that requires renal replacement therapy to sustain life. He is otherwise hemodynamically stable. His wife has medical power of attorney. During previous conversations, his wife has stated a strong desire to continue aggressive therapy and indicated that her best understanding of the patient’s wishes would be to continue with aggressive therapy indefinitely. Now she is insisting on dialysis. Dr. Richards feels that hemodialysis will not alter the patient’s prognosis. He has metastatic cancer and is too unstable to safely receive even palliative chemotherapy. His life expectancy is weeks, and while withholding this life-sustaining treatment could hasten his demise, it will not change the outcome of certain death, as a direct result of complications from his lung cancer. Therefore, Dr. Richards is refusing the dialysis.**

**DIRECTIONS**: In your small group, use cognitive empathy to pursue the View from Everywhere:

**Part A:** Begin with Dr. Richards.

- - What is Dr. Richards’ perspective on the problem?
    - How might Dr. Richards define the conflict?
    - How might Dr. Richards view Mrs. Roberts?

**Part B:** Now turn to Mrs. Roberts.

- - What is Mrs. Roberts’ perspective on the problem?
    - How might Mrs. Roberts define the conflict?
    - How might Mrs. Roberts view Dr. Richards?

**Facilitator’s Discussion Points for the “View from Everywhere”**

- As in the group exercise, the point of the exercise is to have participants attempt to put themselves in the 2 stakeholder’s shoes (Mrs. Roberts and Dr. Richards) to cognitively imagine their different perspectives on the conflict.
- Participants should not be worried about “correct” or “incorrect” answers.
- Experienced mediators understand that without getting a full picture of each stakeholder’s perspective, they are at risk of solving only one part of the problem, so this is an exercise to build the “muscle” of cognitive empathy: how do different people perceive a common situation?
- The first question regarding each of the two stakeholders is a general question so that participants understand the aim of the exercise. The facilitator should introduce the general question and then move to the first of the two subquestions, prompting the respondents with the phrase “For Dr. Richards, the problem is…”
- Ask each participant to complete that first prompt (“For Dr. Richards, the problem is…”).
- The facilitator should then move to the second of the two subquestions, prompting the respondents with the phrase “Dr. Richards views Mrs. Roberts as…” or “Dr. Richards sees Mrs. Roberts as…” or “Dr. Richards considers Mrs. Roberts to be…”
- Each participant should offer an answer to the second prompt.
- After each participant has given a response to each of the two sub questions, the discussion should turn to Mrs. Roberts with the exact same procedure.
  - “For Mrs. Richards, the problem is…”
  - “Mrs. Roberts views Dr. Richards as…” or “Mrs. Roberts sees Dr. Richards as…” or “Mrs. Roberts considers Dr. Richards to be…”
- Finally, ask the group to reflect on those distinct perspectives and ways in which the problem could be identified from both perspectives.
