# Supplementary material for: Using a Mediator's Toolbox: Reducing Clinical Conflict by Learning to Reconceive the “Difficult” Patient or Family
Source: MedEdPORTAL. 2023 Jul 14;19:11324. doi: 10.15766/mep_2374-8265.11324 (PMC10345165; doi:10.15766/mep_2374-8265.11324)
Supplement: Supplementary file 1 — Using the Mediators Toolbox Presentation.pptxView From Everywhere Case Study.docxPositions vs. Interests Case Study.docxWorkshop Evaluation.docx [file mep_2374-8265.11324-s001.zip › C. Positions vs. Interests Case Study.docx]

**Appendix C: Using the Mediator’s Toolbox**

**“Positions vs. Interests” Breakout Session**

**Case Study: Mr. Roberts**

**Case:**

- **John Roberts is a 73-year-old man who presented to the hospital with respiratory distress. He required intubation and mechanical ventilation upon arrival. Subsequently, he underwent a battery of tests that diagnosed metastatic lung cancer. His hospitalization has been complicated by pneumothorax, venous thromboembolism, cardiac arrhythmias, anemia, pneumonia, and severe malnutrition. He has undergone multiple procedures including a tracheostomy and feeding tube placement. One month into his course of treatment, he has persistent respiratory failure and remains ventilator dependent. Additionally, he has now developed acute renal failure that requires renal replacement therapy to sustain life. He is otherwise hemodynamically stable. His wife has medical power of attorney. During previous conversations, his wife has stated a strong desire to continue aggressive therapy and indicated that her best understanding of the patient’s wishes would be to continue with aggressive therapy indefinitely. Now she is insisting on dialysis. Dr. Richards feels that hemodialysis will not alter the patient’s prognosis. He has metastatic cancer and is too unstable to safely receive even palliative chemotherapy. His life expectancy is weeks, and while withholding this life-sustaining treatment could hasten his demise, it will not change the outcome of certain death, as a direct result of complications from his lung cancer. Therefore, Dr. Richards is refusing the dialysis.**

**DIRECTIONS**: In your small group, brainstorm the possible interests that underlie the positions taken by Dr. Richards and Mrs. Roberts:

**Part A:** Begin with Mrs. Roberts. What are Mrs. Roberts’ interests in insisting on dialysis? Use the following prompts:

Mrs. Roberts

- - “is worried about…”
  - “has a need for…”
  - “is concerned about…”
  - “values…”

**Part B:** Now turn to. Dr. Richards. What are Mrs. Roberts’ interests in refusing dialysis? Use the following prompts:

Dr. Richards

- - “is worried about…”
  - “has a need for…”
  - “is concerned about…”
  - “values…”

**Facilitator’s Discussion Points for “Positions vs. Interests”**

- The point of the exercise is to learn how to distinguish the stances that someone takes about a particular issue or choice (often the sources of the conflict) and the underlying reasons why they take the positions they do, i.e., their “interests.”
- Participants should not be worried about “correct” or “incorrect” answers.
- As they are reflecting on what matters to Mrs. Roberts and Dr. Richards, participants should brainstorm what might be possible reasons for the choices they advocate.
- When mediators work with stakeholders, creating a picture of the individual’s worries, needs, concerns, and values enables them to focus the discussion on their “interest” rather than the “positions” that they take on issues and about which they are in conflict.
- Participants should be allowed to use any of the 4 prompts in the exercise.
- Examples for Dr. Richards might be: “Dr. Richards is worried about squandering scarce resources” or “Dr. Richards values modeling judicious use of medical resources.”
- Examples for Mrs. Roberts might be: “Mrs. Roberts values being the best surrogate decision-maker she can be for her husband” or “Mrs. Roberts is worried that the treating team is not doing everything they can to prolong her husband’s life.”
- In an actual conflict management session, the mediator would prompt the stakeholder to reflect on their worries, needs, and concerns.
