# Supplementary material for: Using a Mediator's Toolbox: Reducing Clinical Conflict by Learning to Reconceive the “Difficult” Patient or Family
Source: MedEdPORTAL. 2023 Jul 14;19:11324. doi: 10.15766/mep_2374-8265.11324 (PMC10345165; doi:10.15766/mep_2374-8265.11324)
Supplement: Supplementary file 1 — Using the Mediators Toolbox Presentation.pptxView From Everywhere Case Study.docxPositions vs. Interests Case Study.docxWorkshop Evaluation.docx [file mep_2374-8265.11324-s001.zip › D. Workshop Evaluation.docx]

**Appendix D: Workshop Evaluation**

**DIRECTIONS**: Please take a few moments to provide feedback to the authors for improving this workshop.

Are you currently a student? Yes / No

If yes, what degree are you working on?___________________________

Other Degrees (Choose all that apply):

PhD_____ What field? ________________

JD____

Other masters (MBE,MA, MPH)_____

BA/BS/BSN____

MD_______

**DIRECTIONS**: Circle the response that best matches your opinion. Use the following key:

**SD= Strongly Disagree; D=Disagree; D/A = Disagree/Agree Equally; A=Agree; SA= Strongly Agree**

| 1. This workshop held my interest | **SD** | **D** | **D/A** | **A** | **SA** |
| --- | --- | --- | --- | --- | --- |
| 2. This workshop would be valuable to others in my field | **SD** | **D** | **D/A** | **A** | **SA** |
| 3. I plan to actively use these techniques in working with patients and families in the future | **SD** | **D** | **D/A** | **A** | **SA** |
| 4. As a result of this workshop, I learned information that will help me be better prepared to work with patients and families. | **SD** | **D** | **D/A** | **A** | **SA** |
| 5. If I could decide again, I would still choose to attend this workshop. | **SD** | **D** | **D/A** | **A** | **SA** |
|  | | | | | |
| **As a result of participating in this workshop, I am better able to…** |  |  |  |  |  |
| 6. Diagnose the source of conflicts with families and patients | **SD** | **D** | **D/A** | **A** | **SA** |
| 7. Define positions vs. interests | **SD** | **D** | **D/A** | **A** | **SA** |

**DIRECTIONS**: Circle the response that best matches your opinion. Use the following key:

| **Workshop Components** | **Quality of Module** | | | | |
| --- | --- | --- | --- | --- | --- |
|  | **E=Excellent**  **G=Good**  **S=Satisfactory**  **M=Marginal**  **P= Poor** | | | | |
| 1. Didactic Presentation | **E** | **G** | **S** | **M** | **P** |
| 2. Activity 1: Pursuing the View from Everywhere | **E** | **G** | **S** | **M** | **P** |
| 3. Activity 2: Positions vs. Interests | **E** | **G** | **S** | **M** | **P** |
| 4. Overall workshop evaluation | **E** | **G** | **S** | **M** | **P** |

1. Given the amount of content covered and the activities, rate the length of the session. (Circle one)

**Too Short Just Right Too long**

2. What were the greatest strengths of the workshop?

3. What suggestions do you have to improve the workshop in the future?
