# Supplementary material for: Using a Mediator's Toolbox: Reducing Clinical Conflict by Learning to Reconceive the “Difficult” Patient or Family
Source: MedEdPORTAL. 2023 Jul 14;19:11324. doi: 10.15766/mep_2374-8265.11324 (PMC10345165; doi:10.15766/mep_2374-8265.11324)
Supplement: Supplementary file 1 — Using the Mediators Toolbox Presentation.pptxView From Everywhere Case Study.docxPositions vs. Interests Case Study.docxWorkshop Evaluation.docx [file mep_2374-8265.11324-s001.zip › Educational Summary Report_11324.pdf]

MedEdPORTAL® | The AAMC Journal of Teaching and Learning Resources

Original Publication Open Access

# Using a Mediator's Toolbox: Reducing Clinical Conflict by Learning to Reconceive the "Difficult" Patient or Family

Autumn Fiester, PhD*, Shana Stites, PsyD

*Corresponding author: fiester@upenn.edu

## Abstract

**Introduction:** Given the prevalence of conflict between physicians and patients and families, it is crucial that trainees build the skills to manage clinical conflict. Mediators employ an approach that can be applied to clinical encounters to prevent conflicts from escalating. This workshop introduced trainees to techniques commonly used by mediators to manage disputes. **Methods:** Medical students in a virtual workshop (cohort A) and clinical fellows in an in-person workshop (cohort B) were presented with a mediator's approach to interpreting patient and family behavior viewed as challenging. Trainees were introduced to two specific techniques designed to facilitate the resolution of clinical conflict. After an interactive large-group discussion of each method, small groups practiced applying the technique to a sample clinical case. Finally, participants completed an assessment of their perception of the workshop's effectiveness. **Results:** In early 2022, 15 medical students (cohort A) participated in a virtual workshop and 10 clinical fellows (cohort B) participated in an in-person workshop on clinical conflict management. Eight medical students from cohort A completed the postworkshop assessment (response rate: 53%); six clinical fellows from cohort B completed the assessment (response rate: 60%). Cohort A gave the workshop an overall evaluation of 4.6 out of 5.0; cohort B gave the workshop an overall score of 4.7 out of 5.0. **Discussion:** In both the virtual platform and the traditional in-person format, this workshop introduces a set of tools for navigating bedside conflicts with patients and their families that participants believed would better prepare them for such challenging interactions.

**Keywords:**
Challenging Patient Encounters, Family-Centered Care, Patient-Centered Care, Interpersonal Communication, Mediation, Conflict Management, Communication Skills, Physician-Patient Relationship

## Educational Objectives

By the end of this activity, learners will be able to:

1. Reframe patient and family behavior that is viewed as challenging to enable more constructive collaboration.
2. Identify the sources of provider-patient conflicts by discovering the view from everywhere.
3. Formulate a distinction between positions and interests to refocus the conversation on the underlying reasons for the conflict.

**Citation:**
Fiester A, Stites S. Using a mediator's toolbox: reducing clinical conflict by learning to reconceive the "difficult" patient or family. *MedEdPORTAL*. 2023;19:11324.
https://doi.org/10.15766/mep_2374-8265.11324

## Introduction

Conflict with patients and their families is a pervasive problem in clinical practice for providers. Research on physician-patient interactions indicates that 15%-60% of patients are considered difficult by their clinical providers.[1-3] While that is a broad range, it represents a substantial portion of patients, even at its lower end. Many clinical encounters are fraught with disruptive conflict, enervating interactions, and intense frustration. Patient and family behaviors often include discourtesy, verbal abuse, and offensive language. Patient and family behaviors that are considered challenging are not limited to abusive language. Clinical teams often experience threats of physically aggressive behavior, and reports of violence in the clinic are becoming more common.[4] There is a robust literature demonstrating the consequences of clinical burnout and psychological distress generated by these dysfunctional interactions.[5-8]

Given the prevalence and impact of the problems caused by challenging clinical interactions, physicians and trainees may

Copyright © 2023 Fiester and Stites This is an open-access publication distributed under the terms of the Creative Commons Attribution license. 

benefit from learning conflict management skills.[9-16] Although physicians in every specialty might benefit from mediation techniques, there may be a heightened need in high-intensity units such as the ICU, emergency room, and psychiatric settings.[17-20] While some publications describe verbal and nonverbal communication techniques, conflict de-escalation approaches, and skills for managing patient anger that can assist clinicians with challenging clinical encounters,[12,14,16,21-22] they are very brief and succinct descriptions of approaches fitting within the parameters of a typical-length medical journal article. Few educational materials offer clinicians step-by-step training workshops in specific mediation techniques that can address challenging physician-patient conflict.[23-25] Three recent training workshops in *MedEdPORTAL* provide structured resources for learning mediation techniques, each focusing on discrete aspects of conflict management to address different physician concerns. One centers on conflicts among providers from different disciplines and/or different stages of training to assist in navigating power hierarchies.[23] Relatedly, a second training module, recognizing gender as a factor in power dynamics, is designed to empower women in health care (nurses, physicians, trainees) to communicate effectively and build productive teams in order to improve their wellness.[24] A third focuses directly on patient-provider conflicts, borrowing insights from business management settings, with an emphasis on articulating shared goals, clarifying respective roles in patient-provider interactions, and assessing the quality of the interpersonal skills needed for productive interactions.[25]

We developed our resource to introduce physicians to a facet of conflict management that has not been addressed by the other resources, namely, the task of accurately determining the precise nature of the conflict. For 40 years,[26] mediators have understood that one of the most important steps in effective conflict management is framing the problem in a way that aids constructive conflict resolution. This involves conceptualizing the problem so as not to sabotage opportunities for successful intervention. Research on the “difficult” patient demonstrates that physicians frequently conceptualize the problem of disruptive patients exclusively from their own clinical perspective.[27,28] The most prevalent explanatory framework for the “difficult” patient is the presence of a psychiatric disorder. One early study on the “difficult” patient concluded, “The difficult or frustrating patient … often has unrecognized psychiatric problems.”[28] Another study about physicians’ perspective on patients considered challenging listed a range of psychiatric diagnoses as the explanation for the disruptive behavior including “comorbid psychopathology, hostility, suicidality, aberrant drug behavior, and chronic noncompliance.”[29] From a mediation perspective, this way of defining the problem of challenging interactions undermines a mediator’s ability to improve patient-provider dynamics and generate more satisfying clinical interactions. Because interactions are interpersonal, that is, between people, the definition of a problem must be multiperspectival, rather than one-sided. Otherwise, the mediator will only be able to solve one party’s problem rather than the problem that exists between the parties. Some physician research teams have begun to employ this insight from the field of conflict management, calling for a better understanding of the patient’s or family’s perspective in the service of improving strained doctor-patient relationships.[10,30-32] The literature provides examples of physician behaviors that can engender patient-provider or family-provider conflict, such as poor communication, interaction styles viewed as arrogant or patronizing, perceived bias or discrimination, and paternalism in medical decision-making.[32-34]

Conflict management specialists view the imperative to understand the perspective of all parties in the conflict as part of the conflict diagnosis.[9,26] Adequate conflict diagnosis includes investigating how various stakeholders understand the problem, its scope, and its sources. Untangling the misunderstandings, mischaracterizations, and misperceptions of the stakeholders is essential for an accurate conflict diagnosis. Grasping the reasons why the parties take the stances and exhibit the behavior they do is of paramount importance for the prospects of attitudinal and behavioral change. Only brief descriptive training resources exist for physicians and trainees in this specific aspect of conflict analysis,[9,15,16] though conflict management experts agree on the need for mastery of conflict assessment.[9,26]

Our workshop offers specific training in how to avoid a flawed conflict analysis by providing step-by-step instruction in two techniques employed by mediators to secure an accurate conflict diagnosis: (1) ascertaining the view from everywhere and (2) distinguishing between positions and interests. Both these conflict management tools have the potential to enhance clinicians’ ability to remediate dysfunctional clinical relationships and mitigate patient or family behavior that is considered challenging. These two devices have the potential to enable health care providers to troubleshoot conflict as it is emerging and potentially to reduce the frequency of conflicts occurring in the first place. While our workshop was offered to trainees, this training could be valuable to clinicians at different stages of practice. These conflict management skills have the potential to reduce workplace stress and enhance professional satisfaction.

Copyright © 2023 Fiester and Stites This is an open-access publication distributed under the terms of the Creative Commons Attribution license.

While the training in our module could be helpful to a clinician in dealing with patients or families viewed as challenging (or in preventing conflict from escalating in the first place), there might be times when it would be necessary to have third-party mediator work with the parties to resolve very entrenched or inflamed conflicts. A hospital's ethics consultation service frequently has the resources to assist clinicians in such circumstances.[13] Additionally, our workshop offers a process for managing conflict that arises from differences in perception or opinion between patients, families, and providers, but this approach is not appropriate for instances where there is an altered mental status (e.g., intoxication or psychosis) in the patient or family.

## Methods

Borrowing the conceptual framework first developed by Fisher and Ury in the Harvard Negotiation Project in 1981,[26] we introduced core concepts in mediation theory to offer a tool for conflict assessment. The workshop was implemented on two occasions with different participants (cohort A and cohort B), using different platforms (virtual and in person). The workshop for cohort A was an elective for fourth-year medical students during a mandatory weeklong intensive in medical ethics at a mid-Atlantic medical school; it was conducted virtually. Participants in cohort B were clinical fellows from a wide variety of clinical specialties who were enrolled in a small master's program in health policy in a mid-Atlantic academic medical center; it was conducted in person. Although the two iterations of the workshop differed by learner groups (medical students vs. fellows), length (60 minutes vs. 90 minutes), and format (in person vs. virtual), the workshops were essentially the same in structure and content. The key differences were the amount of time participants had available for the group exercises and the absence of breakout sessions in cohort A. The reason why the workshops had identical content despite differences in learner groups was that none of the participants in either cohort had any prior experience with conflict management training, despite significant differences in medical training and experience. The workshop was designed for conflict management novices, and participants in both groups were beginners.

For cohort A, we presented the workshop in a 1-hour, synchronous, virtual format with no small-group breakout sessions. The workshop was originally planned to be in person, but due to an increase in cases of COVID-19, all classes at the medical school were moved to an online platform shortly before the course took place. Because of the change in platform, the workshop was shortened, and small-group breakout sessions were not possible due to the time constraints. Fifteen students in the fourth-year medical school class chose to take the extra elective. For cohort B, we presented the workshop in a 90-minute in-person format with two small-group breakout sessions. Our workshop was conducted during one of the regularly scheduled professional development sessions. Ten clinical fellows participated in the second workshop. The students did not need any prior background or preparation for the workshop.

The facilitator for both workshops was a medical ethics scholar and expert in conflict management with hands-on experience in mediating conflict, as well as substantial experience training health care providers in mediation techniques. Although the workshop facilitator had extensive experience as a trainer in conflict management techniques, the educational materials for the workshop (the PowerPoint presentation and handouts) were designed to be used by facilitators with no conflict management expertise. The materials were created to function as a group-guided modality for students motivated to master new techniques for use in clinical practice. The target audience was medical trainees at two different stages of training: medical students and fellows.

The workshop was designed to be used with small-group breakout sessions, but the small-group breakout sessions for cohort A had to be omitted when the workshop was moved to the virtual platform due to COVID-19. Both workshops used the presentation in Appendix A. In the in-person workshop, the facilitator assigned three small groups of five participants each. In the in-person setting, handouts (Appendices B-D) were printed prior to the workshop and distributed at the appropriate time. Each participant was given a copy of each handout for the breakout session (Appendices B and C). A cue for the timing to distribute the appropriate handout was embedded in the PowerPoint presentation. The workshop assessment (Appendix D) was distributed at the end of the session and then collected.

In the virtual platform, there were no small groups, so Appendices B and C were not used. The workshop assessment (Appendix D) was linked through the Chat function and, once completed, scanned or emailed back to the facilitator.

For the in-person setting, the workshop included the following materials:

- Audiovisual equipment (computer and projector)
- PowerPoint presentation (Appendix A)
- Handouts (Appendices B-D)

Copyright © 2023 Fiester and Stites This is an open-access publication distributed under the terms of the Creative Commons Attribution license.

- Pens (to fill out the workshop assessment [Appendix D] at the end of the workshop)
- Table and chairs set up to accommodate the desired number and size of the small-group breakout sessions

Workshop Structure

Workshops were structured as follows:

- Introduction (Appendix A, slides 1-2): introduction of the facilitator, review of the session's objectives (5 minutes). The facilitator gave a self-introduction. Attendees in both cohorts A and B already knew each other, so introductions were not necessary. Participants were provided with an overview of the workshop's objectives.
- Stage Setting: The "Difficult" Patient (Appendix A, slides 3-8): background context and research on the "difficult" patient (10 minutes). The facilitator provided an overview of the research on patients viewed as disruptive or challenging in the clinical context, including the negative consequences on providers, conventional interpretations of the cause of disruptive behavior, recent scholarship challenging the conventional view of the "difficult" patient, rationale for getting accurate conflict diagnosis, and the introduction of the mediator's toolbox.
- Technique 1: View From Everywhere (Appendix A, slides 9-24): description of the first technique in the mediator's toolbox, ascertaining the view from everywhere (10 minutes). The facilitator introduced the case study employed throughout the session. Then, the facilitator described the first step in generating an accurate conflict diagnosis: pursuing the view from everywhere.
- Group Activity 1: View From Everywhere (Appendix A, slides 20-22): all-group practice in determining the perspectives of all stakeholders (10 minutes). The facilitator led a large-group discussion using the tool of cognitive empathy to uncover the perspectives of the main stakeholders in the conflict.
- Small-Group Activity 1: View From Everywhere (Appendix A, slides 23-25; Appendix B): practice employing technique 1 in breakout groups of two to five, using the case study of Appendix B (10 minutes). The facilitator introduced the case study for the small-group exercises and distributed Appendix B. In their small groups, participants used the tool of cognitive empathy to distill the perspectives of the two parties in the case study conflict. (Omitted with cohort A.)
- Technique 2: Positions vs. Interests (Appendix A, slides 27-33): description of distinguishing between positions and interests, the second technique in the mediator's toolbox (10 minutes). The facilitator described the second step in generating an accurate conflict diagnosis: distinguishing between a stakeholder's positions and their interests.
- Group Activity 2: Positions vs. Interests (Appendix A, slides 34-38): all-group practice in determining the interests of the stakeholders involved in the workshop case study (10 minutes). Using the case study introduced in technique 1, the facilitator led an all-group exercise, with prompts presented on slides 42 and 44, in determining the possible interests of the conflicting parties.
- Small-Group Activity 2: Positions vs. Interests (Appendix A, slides 39-40; Appendix C): practice employing technique 2 in breakout groups of two to five, using the case study of Appendix C (10 minutes). The facilitator reintroduced the case study used in the first small-group exercises and distributed Appendix C with the prompts for the breakout session discussion. In their small groups, participants used the prompts in Appendix C to determine the possible interests of the two parties in the conflict. (Omitted with cohort A.)
- Workshop Wrap-up (Appendix A, slide 41): review of workshop takeaways, Q&A, and list of references (15 minutes). The facilitator gave an overview of the concepts and techniques learned in the workshop and reviewed the references used in the presentation so that participants could explore the relevant literature. The facilitator used the remaining time for questions from and discussion with the attendees.

Assessment

Participants completed a workshop assessment that was distributed at the end of the session. For both cohorts A and B, attendees were emailed a link to the survey by the session organizers (not the facilitator/authors) following the workshop. The platform utilized for the workshop assessment was Qualtrics. Participants were emailed the survey by the session organizers at the request of the institutional review board to provide complete anonymity for survey respondents. Participants were informed at the beginning of the workshop that they would be asked to complete a voluntary survey delivered by email at the session's conclusion. Informed consent was obtained through language on the actual survey. We obtained University of Pennsylvania Institutional Review Board approval for this workshop through expedited review; the workshop was considered not to be human research.

The assessment (Appendix D) included 14 questions. Seven captured the attendee's impression of the workshop, four evaluated specific segments and then the overall quality of the

Copyright © 2023 Fiester and Stites This is an open-access publication distributed under the terms of the Creative Commons Attribution license.

workshop, two were open-ended qualitative questions about the workshop's strengths and weaknesses, and one asked about workshop length. Attendees responded to each of the seven impression questions on a 5-point Likert scale (1 = *strongly disagree,* 5 = *strongly agree*) and evaluated the workshop on another 5-point scale (1 = *poor,* 5 = *excellent*). No demographic data were collected except current and past degrees.

## Results

In early 2022, 15 medical students (cohort A) participated in a virtual workshop and 10 clinical fellows (cohort B) participated in an in-person workshop on clinical conflict management. All participants in each cohort completed the respective workshop for completion rates of 100%. Eight of the 15 medical students from cohort A completed the postworkshop assessment for a response rate of 53%. Six of the 10 clinical fellows from cohort B completed the assessment for a response rate of 60%.

Participants from cohort A gave the workshop an overall mean evaluation of 4.6 out of 5.0 points. Those from cohort B gave the workshop an overall mean rating of 4.7 out of 5.0 points (Table). If given another chance to choose whether to attend the workshop, most participants in cohorts A and B said they would again choose to attend (mean ratings of 4.4 out of 5.0 points and 4.5 out of 5.0 points, respectively). In addition, most participants found the length of the workshop to be satisfactory, with 75% of cohort A and 83% of cohort B reporting the length of the workshop session to be "just right."

Participants in cohorts A and B felt the workshop would be valuable to others in their field (mean ratings of 4.9 out of 5.0 and 4.8 out of 5 .0, respectively). Participants in cohorts A and B planned to use the techniques in working with patients and families in the future (mean ratings of 4.6 out of 5.0 and 4.7 out of 5.0, respectively). Participants in cohorts A and B also felt better able to diagnose the source of conflicts with families and patients (mean ratings of 4.1 out of 5.0 and 4.7 out of 5.0, respectively).

Responses to the open-ended item that asked participants to list areas of improvement for the workshop included recommendations to add a one-page tips sheet, which would feature suggestions from the workshop and sample language, and to lengthen the duration of the workshop to 1.5 hours instead of 1 hour in order to be able to engage in deeper discussion of concepts. Responses to the open-ended item that asked participants to list strengths of the workshop included the use of real-world examples, perception that the recommendations were actionable, and interactive teaching components.

## Discussion

This workshop was developed after a review of the existing training resources for physicians working with challenging patients. There is a paucity of curricula that focus on step-by-step training in conflict management on this topic. Trained mediators have a multitude of techniques and approaches that can enable stakeholders to avoid conflict, prevent conflicts from escalating, and de-escalate heated interactions. The goal of this workshop was to provide clinicians and trainees with a set of basic tools from the mediator's toolbox that could be implemented to help mitigate and even prevent challenging clinical encounters, focusing on the crucial first step of mediation, namely, accurately identifying the problem from the perspective of all parties.

**Table.** Summary of Postworkshop Survey Results

| | Cohort A (*N* = 8) | | Cohort B (*N* = 6) | |
|---|---|---|---|---|
| Item | *M* (*SD*) | No. (%) | *M* (*SD*) | No. (%) |
| This workshop held my interest | 4.5 (0.5) | | 4.7 (0.8) | |
| This workshop would be valuable to others in my field | 4.9 (0.3) | | 4.8 (0.4) | |
| I plan to actively use these techniques in working with patients and families in the future | 4.6 (0.5) | | 4.7 (0.8) | |
| As a result of this workshop, I learned information that will help me be better prepared to work with patients and families | 4.5 (0.5) | | 4.5 (0.8) | |
| If I could decide again, I would still choose to attend this workshop | 4.4 (0.7) | | 4.5 (0.8) | |
| As a result of this workshop, I am better able to diagnose the source of conflicts with families and patients | 4.1 (0.8) | | 4.7 (0.5) | |
| As a result of this workshop, I am better able to define positions versus interests | 4.4 (0.7) | | 4.8 (0.4) | |
| Didactic presentation | 4.5 (0.5) | | 4.8 (0.4) | |
| Activity 1: Pursuing the View From Everywhere[a] | 4.3 (0.7) | | 4.8 (0.4) | |
| Activity 2: Positions vs. Interests[a] | 4.4 (0.7) | | 5.0 (0.0) | |
| Overall workshop evaluation | 4.6 (0.5) | | 4.7 (0.5) | |
| Given the amount of content covered and the activities, rate the length of the session | | | | |
| Too short | | 2 (25) | | 0 (0) |
| Just right | | 6 (75) | | 5 (83) |
| Too long | | 0 (0) | | 1 (17) |

[a]One person had missing data.

Copyright © 2023 Fiester and Stites This is an open-access publication distributed under the terms of the Creative Commons Attribution license.

We found that the two core techniques were perceived by attendees to be of potential help in working with patients based on the very high scores from cohorts A and B. Both cohorts planned to use these techniques in working with patients and families, and they believed they were better prepared to work with patients and families in the future. We found that the workshop was adaptable to both virtual and in-person settings. We also found that the workshop was adaptable for use with small-group breakout sessions and with merely large-group discussion.

Though the group sizes were small, there may have been an identifiable trend in the ratings indicating a preference for a 90-minute session with breakout groups over the shorter 60-minute all-group session. The shorter virtual workshop (cohort A) had slightly lower evaluations than did the longer in-person session (cohort B). This difference in ratings could potentially have been due to the difference in the workshop platform (virtual vs. in person) or duration (60 minutes vs. 90 minutes) or to the use of breakout groups versus an all-group format. We do not have the data to tease apart these variables, but the facilitator perceived the shorter workshop as rushed, which might indicate a need for the extra 30 minutes. The trainees in cohort A did not get the additional practice that cohort B received through the breakout sessions, which we believe added value. In running future workshops, we would schedule the workshops for 90 minutes and include the breakout sessions. Additionally, while a majority of participants in cohort A viewed the length of the workshop as just right, a 1-hour workshop may be too short in the absence of an expert facilitator. A less experienced trainer may require more time to achieve the goals of the workshop.

The difference in the workshop evaluations between cohorts A and B might also be explained by the career-stage of the participants. Cohort A comprised fourth-year medical students who would have had limited experience managing complex patient and family interactions on their own without direct supervision from their residents, fellows, and attendings. Cohort B comprised clinical fellows who, though still early in their career, would have had more years of experience in managing disruptive patients and families throughout their training.

In both workshops, participation levels were high, with most participants actively engaging in the discussion and exercises, despite the difference in format (in person vs. virtual). We believe that the virtual format would pose additional challenges, however, if the group were large. With only 15 participants, the facilitator could see all the students on one screen. This enabled the facilitator to monitor participation levels and actively engage the more reticent students. If the group were larger, the facilitator would not be able to view all members of the workshop at once, which would reduce the facilitator's ability to ensure equal participation. We recommend that virtual iterations of the workshop remain small enough so that all participants can be seen on one screen.

While we did not include breakout sessions in the shorter virtual workshop, we do not believe that this format precludes breakout groups. Breakout groups can easily be assigned automatically through the functions embedded in the common virtual platforms (e.g., Zoom, Microsoft Teams, BlueJeans). In a virtual platform, the participants could access the needed handouts (Appendices B and C) through the Chat function via a link connecting to a platform like Box or Dropbox. The facilitator could create this link prior to the workshop and post it at appropriate times during the session.

Our project was limited by the small number of participants, and so, results cannot be generalized. We piloted this workshop with medical students and clinical fellows; therefore, we do not know how effective it would be with residents or attendings. While there is overlap in the evaluations between cohort A (medical students) and cohort B (fellows), we do not know if residents would have a response similar to that of our participants, given the different challenges and pressures residents face. Although we anticipate that attendings could benefit from this workshop, it is possible they may have already been exposed to conflict management training and therefore would already have the core skill set we teach.

Future work could expand the number of participants in the workshop to achieve a larger sample size for assessment. We collected no data from cohort B about medical specialty, and future work might explore the possibility that different clinical specialties have different responses and different needs with regard to conflict management training. Future work could also measure the efficacy of this workshop with participants from a wider setting, including the two groups omitted in our pilot studies, residents and attendings. Because conflicts can occur in all health care settings, future work could customize the training modules to particular types of providers and modify the conflicts employed to be relevant to those subspecialties. Future work could also design an assessment that could evaluate the acquired skills or behavior change generated by the workshop. A future training module could use a video clip of actors modeling various aspects of the conflict management process, including how the points of view of each stakeholder are solicited and the way in which a skilled facilitator identifies the parties' interests. A

Copyright © 2023 Fiester and Stites This is an open-access publication distributed under the terms of the Creative Commons Attribution license.

video clip could also be useful in depicting the conflict itself. The field of conflict management employs a vast array of techniques to resolve conflict, and future work could develop workshops focusing on additional, more advanced mediation skills. For example, a future training module could also include simulations (perhaps using actors) so that attendees could employ these techniques in mock conflict settings. Pre- and posttraining evaluations could assess the efficacy of the the skill-building from these simulations.

Our workshop does not address strategies for specifically dealing with language or behavior that is racist, homophobic, sexist, anti-Semitic, and so on. These types of hurtful and offensive interactions present complex challenges, and a future training model could be designed to focus exclusively on this important subset of conflict management. Similarly, our workshop does not include training on cultural competency, trauma-informed care, or historical legacies of marginalized health care populations and mistrust of health care systems, all of which can contribute to physician-patient conflict. A future direction could be to build a module that provides training in these important health care contexts.

This project demonstrates that medical trainees perceive potential value in being introduced to two core techniques from the mediator's toolbox, the view from everywhere and positions versus interests, through a brief training session. The pervasiveness of challenging clinical encounters establishes a clear need for conflict management training. The evaluations of this workshop suggest that teaching core mediation techniques could provide potential tools for trainees in managing these challenging clinical situations.

## Appendices

A. Using the Mediators Toolbox Presentation.pptx

B. View From Everywhere Case Study.docx

C. Positions vs. Interests Case Study.docx

D. Workshop Evaluation.docx

*All appendices are peer reviewed as integral parts of the Original Publication.*

**Autumn Fiester, PhD:** Associate Professor, Department of Medical Ethics and Health Policy, Perelman School of Medicine at the University of Pennsylvania; Director, Program in Clinical Conflict Management, University of Pennsylvania; ORCID: https://orcid.org/0000-0002-2087-0826

**Shana Stites, PsyD:** Assistant Professor, Department of Psychiatry, Perelman School of Medicine at the University of Pennsylvania

### Disclosures
None to report.

### Funding/Support
None to report.

### Prior Presentations
Fiester A. Working with the "difficult" patient or family. Presented virtually at: Bioethics Master Program Colloquium, Duquesne University; May 6, 2021.

### Ethical Approval
The University of Pennsylvania Institutional Review Board deemed further review of this project not necessary.

## References

1. Hahn SR, Thompson KS, Wills TA, Stern V, Budner NS. The difficult doctor-patient relationship: somatization, personality and psychopathology. *J Clin Epidemiol.* 1994;47(6):647-657. https://doi.org/10.1016/0895-4356(94)90212-7
2. Jackson JL, Kroenke K. Difficult patient encounters in the ambulatory clinic: clinical predictors and outcomes. *Arch Intern Med.* 1999;159(10):1069-1075. https://doi.org/10.1001/archinte.159.10.1069
3. Hahn SR. Physical symptoms and physician-experienced difficulty in the physician–patient relationship. *Ann Intern Med*. 2001;134(9 pt 2):897-904. https://doi.org/10.7326/0003-4819-134-9_Part_2-200105011-00014
4. Devnani M. Covid-19 and laws for workplace violence in healthcare. *BMJ*. 2021;375:n2776. https://doi.org/10.1136/bmj.n2776
5. Nowrouzi B, Lightfoot N, Larivière M, et al. Occupational stress management and burnout interventions in nursing and their implications for healthy work environments: a literature review. *Workplace Health Saf.* 2015;63(7):308-315. https://doi.org/10.1177/2165079915576931
6. West CP, Dyrbye LN, Shanafelt TD. Physician burnout: contributors, consequences and solutions. *J Intern Med*. 2018;283(6):516-529. https://doi.org/10.1111/joim.12752
7. Fitzpatrick B, Bloore K, Blake N. Joy in work and reducing nurse burnout: from triple aim to quadruple aim. *AACN Adv Crit Care*. 2019;30(2):185-188. https://doi.org/10.4037/aacnacc2019833
8. Dall'Ora C, Ball J, Reinius M, Griffiths P. Burnout in nursing: a theoretical review. *Hum Resour Health*. 2020;18:41. https://doi.org/10.1186/s12960-020-00469-9
9. Bergman EJ. Identifying sources of clinical conflict: a tool for practice and training in bioethics mediation. *J Clin Ethics.*

Copyright © 2023 Fiester and Stites This is an open-access publication distributed under the terms of the Creative Commons Attribution license. 

2015;26(4):315-323. https://doi.org/10.1086/JCE2015264315

10. Blackall GF, Green MJ. “Difficult” patients or difficult relationships? *Am J Bioeth*. 2012;12(5):8-9. https://doi.org/10.1080/15265161.2012.665137
11. Cochran N, Charlton P, Reed V, Thurber P, Fisher E. Beyond fight or flight: the need for conflict management training in medical education. *Conflict Resolut Q*. 2018;35(4):393-402. https://doi.org/10.1002/crq.21218
12. Pluhar E, Power S, Freizinger M, Altman W. Medical education: guidelines for effective teaching of managing challenging patient encounters. *Med Sci Educ*. 2019;29(3):855-861. https://doi.org/10.1007/s40670-019-00729-x
13. Fiester A. The “difficult” patient reconceived: an expanded moral mandate for clinical ethics. *Am J Bioeth*. 2012;12(5):2-7. https://doi.org/10.1080/15265161.2012.665135
14. Fiester AM. What mediators can teach physicians about managing “difficult” patients. *Am J Med*. 2015;128(3):215-216. https://doi.org/10.1016/j.amjmed.2014.09.017
15. Fiester A. The “ladder of inference” as a conflict management tool: working with the “difficult” patient or family in healthcare ethics consultations. *HEC Forum*. Published online April 18, 2022. https://doi.org/10.1007/s10730-022-09476-w
16. Saltman DC, O’Dea NA, Kidd MR. Conflict management: a primer for doctors in training. *Postgrad Med J*. 2006;82(963):9-12. https://doi.org/10.1136/pgmj.2005.034306
17. Bowers L. Safewards: a new model of conflict and containment on psychiatric wards. *J Psychiatr Ment Health Nurs*. 2014;21(6):499-508. https://doi.org/10.1111/jpm.12129
18. Morreim H. Mediating healthcare disputes more, earlier … and differently: mediating directly in the clinical setting. *Health Lawyer*. 2018;31(1):18-29.
19. Kayser JB, Kaplan LJ. Conflict management in the ICU. *Crit Care Med*. 2020;48(9):1349-1357. https://doi.org/10.1097/CCM.0000000000004440
20. Bosslet GT, Pope TM, Rubenfeld GD, et al; American Thoracic Society ad hoc Committee on Futile and Potentially Inappropriate Treatment. An official ATS/AACN/ACCP/ESICM/SCCM policy statement: responding to requests for potentially inappropriate treatments in intensive care units. *Am J Respir Crit Care Med*. 2015;191(11):1318-1330. https://doi.org/10.1164/rccm.201505-0924ST
21. Feudtner C. Collaborative communication in pediatric palliative care: a foundation for problem-solving and decision-making. *Pediatr Clin North Am*. 2007;54(5):583-607. https://doi.org/10.1016/j.pcl.2007.07.008
22. Moore PM, Rivera Mercado S, Grez Artigues M, Lawrie TA. Communication skills training for healthcare professionals working with people who have cancer. *Cochrane Database Syst Rev*. 2013;(3):CD003751. https://doi.org/10.1002/14651858.CD003751.pub3
23. Shrader S, Zaudke J. Crucial conversations and conflict resolution interprofessional simulation. *MedEdPORTAL*. 2015;11:10063. https://doi.org/10.15766/mep_2374-8265.10063
24. Welch J, Jimenez H, Allen S. Teamwork & conflict management workshop. *MedEdPORTAL*. 2013;9:9605. https://doi.org/10.15766/mep_2374-8265.9605
25. Wolfe AD, Hoang KB, Denniston SF. Teaching conflict resolution in medicine: lessons from business, diplomacy, and theatre. *MedEdPORTAL*. 2018;14:10672. https://doi.org/10.15766/mep_2374-8265.10672
26. Fisher R, Ury W. *Getting to Yes: Negotiating Agreement Without Giving in.* Penguin; 1983.
27. Kahn MW. What would Osler do? Learning from “difficult” patients. *N Engl J Med*. 2009;361(5):442-443. https://doi.org/10.1056/NEJMp0902021
28. Haas LJ, Leiser JP, Magill MK, Sanyer ON. Management of the difficult patient. *Am Fam Physician*. 2005;72(10):2063-2068.
29. Wasan AD, Wootton J, Jamison RN. Dealing with difficult patients in your pain practice. *Reg Anesth Pain Med*. 2005;30(2):184-192. https://doi.org/10.1016/j.rapm.2004.11.005
30. Gunderman RB, Gunderman PR. Forty years since “Taking Care of the Hateful Patient.” *AMA J Ethics*. 2017;19(4):369-373. https://doi.org/10.1001/journalofethics.2017.19.4.nlit1-1704
31. Strous RD, Ulman AM, Kotler M. The hateful patient revisited: relevance for 21st century medicine. *Eur J Intern Med*. 2006;17(6):387-393. https://doi.org/10.1016/j.ejim.2006.04.002
32. Elder N, Ricer R, Tobias B. How respected family physicians manage difficult patient encounters. *J Am Board Fam Med*. 2006;19(6):533-541. https://doi.org/10.3122/jabfm.19.6.533
33. Krebs EE, Garrett JM, Konrad TR. The difficult doctor? Characteristics of physicians who report frustration with patients: an analysis of survey data. *BMC Health Serv Res*. 2006;6:128 https://doi.org/10.1186/1472-6963-6-128
34. Hull SK, Broquet K. How to manage difficult patient encounters. *Fam Pract Manag*. 2007;14(6):30-34.



**Received:** September 24, 2022
**Accepted:** April 6, 2023
**Published:** July 14, 2023


Copyright © 2023 Fiester and Stites This is an open-access publication distributed under the terms of the Creative Commons Attribution license.
